# Salt-rich versus salt-poor structural scenarios in the central Northern Calcareous Alps: implications for the Hallstatt facies and early Alpine tectonic evolution (Eastern Alps, Austria)

**DOI:** 10.1007/s00531-023-02377-4

**Published:** 2024-01-26

**Authors:** Oscar Fernandez, Hugo Ortner, Diethard Sanders, Bernhard Grasemann, Thomas Leitner

**Affiliations:** 1 Department of Geology, University of Vienna, Josef-Holaubek-Platz 2 (UZA II), 1090 Vienna, Austria; 2 Department of Geology, University of Innsbruck, Innrain 52f, 6020 Innsbruck, Austria; 3 Salinen AG, Altaussee 139, 8992 Altaussee, Austria

**Keywords:** Eastern Alps, Cross-section, Thrust tectonics, Salt tectonics, Gravitational gliding, Structural uncertainty

## Abstract

One of the most remarkable features of the central Northern Calcareous Alps (Eastern Alps, Austria) is the widespread presence of Upper Triassic deep-water carbonates (the Hallstatt facies) and Permo-Triassic evaporites resting on deep-water Middle Jurassic strata and their underlying Upper Triassic shallow-water carbonate platform successions. The Hallstatt facies and accompanying evaporites have been classically interpreted to originate either from a location south of the time-equivalent carbonate platforms, or to have been deposited in deeper water seaways within the broad platform domain. To date, this dispute has been addressed mostly through the analysis of Triassic and Jurassic facies distribution in map view, which, however, is subject to some degree of ambiguity and subjectivity. In this contribution we present, for the first time, sequentially restored regional cross-sections through the central Northern Calcareous Alps to understand the implications of the contrasting paleogeographic models. We present (a) an interpretation based on a highly allochthonous origin of the Triassic deep-water units and (b) an interpretation based on their relative autochthony in which we incorporate the potential influence of salt tectonics in the central NCA. The restored cross-sections provide a framework within which the alternative scenarios and their paleogeographic implications can be better understood. Through this analysis we propose that salt tectonics in the central NCA can provide a valid explanation for apparent inconsistencies in the relative autochthony scenario and thus constitutes a reasonable alternative to the currently accepted allochthony scenario.

**Graphical abstract:**

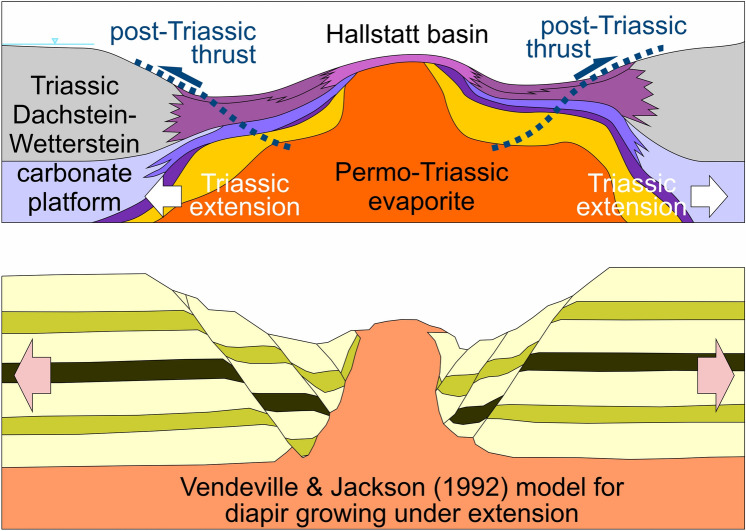

## Introduction

Tectono-sedimentary relationships are key indicators to unravel the tectonic evolution of fold-and-thrust belts. The Northern Calcareous Alps (NCA) of the Eastern Alps of Austria are no exception, and such relationships for Mesozoic strata have been widely used to understand their kinematics (e.g., Peresson and Decker 1997; Faupl and Wagreich 2000; Frisch and Gawlick 2003; Schweigl and Neubauer 1997; Frank and Schlager 2006; Froitzheim et al. 2008; Ortner et al. 2008; Gawlick and Missoni 2019). Consensus exists that sedimentary rocks deposited in the NCA since the Late Jurassic reflect the initiation and progression of the Alpine Orogeny (e.g., Faupl and Wagreich 2000; Frisch and Gawlick 2003). In spite of this general agreement, there still exist diverging views on the pre-Jurassic paleogeography of the NCA, and on the precise nature of Late Jurassic to Early Cretaceous tectonism. The alternative hypotheses have been summarized and discussed, among others, by Faupl and Wagreich (2000), Mandl (2000) and Frisch and Gawlick (2003).

Most of the conflicting paleogeographic reconstructions of the NCA, and the attempts at understanding their Jurassic to Cretaceous structural evolution, have been driven by map-based analysis of facies distributions of unit both pre-dating and synchronous to Late Jurassic to Early Cretaceous tectonism (e.g., Zankl 1967; Schlager 1967; Tollmann 1981; Haas et al. 1995; Schweigl and Neubauer 1997; Gawlick et al 1999; Mandl 2000; Frisch and Gawlick 2003). The main controversy hinges on disagreements on the significance of the present-day distribution and on the inferred original distribution of Upper Triassic facies belts. In particular, the disagreement centers on the origin of a suite of Triassic deposits that are grouped informally under the term “Hallstatt facies” (and which will be referred to hereafter as the Hallstatt units). The Hallstatt units are assemblages of predominantly Upper Triassic deep-water strata, up to a few hundreds of meters in thickness. The Hallstatt units can be encountered today in diverse structural relationships with their time-equivalent platform carbonates, but most commonly they are thrust onto the latter. Hallstatt unit occurrences are typically associated with outcrops of Permo-Triassic evaporites and clastics, and of Middle to Upper Jurassic syntectonic deposits coeval with emplacement of the Hallstatt units.

The contrast in the depositional environments of Upper Triassic carbonates has been known for over a century and already led Hahn (1913a, b) to identify different tectonic units supported by facies differences. Following from the seminal work of Hahn, different interpretations have been put forward since then for the origin of the deep-water Hallstatt units. Hypotheses that seek to explain the observed structural relationships between the Hallstatt units and adjacent units can be placed in two groups. One set of hypotheses postulates a single Hallstatt facies belt deposited in a distal shelf setting with Triassic diapirism, and located south of the shallow-water platform domain. The Hallstatt units were later emplaced onto the platform domain by northward transport, implying a significant allochthony of the Hallstatt units (e.g., Tollmann 1981; Mandl 2000; Gawlick and Missoni 2019). A second set of hypotheses interprets that the Hallstatt units accumulated in deep-water seaways within the shallow-water carbonate platform (e.g., Schlager 1967; Häusler 1980). This implies that the Hallstatt units required limited transport to be thrust onto their neighboring time-equivalent shallow-water carbonates, and are therefore relatively autochthonous.

To date, however, the precise nature of the structural relationships between the Hallstatt units and their adjacent Triassic and Jurassic units has not been studied systematically. Essentially, there have been to date, no regional-scale cross-sections of the central NCA aiming to represent or analyze these relationships in detail. In this contribution we present, for the first time, regional cross-sections through three of the main accumulations of Hallstatt units in the central NCA, constrained by field work and 1:50,000 scale mapping. The aim is to evaluate the structural implications of the aforementioned hypotheses with the techniques of modern structural geology. The sections we present include two alternative interpretations, corresponding to concepts of relative autochthony or of allochthony of the Hallstatt units. These cross-sections are restored sequentially, the first such exercise ever presented for the central NCA, to discuss the implications of structural interpretation on the paleogeography of the Hallstatt units and on the structure of the broader NCA in general.

## Geological setting and background

**Fig. 1:**
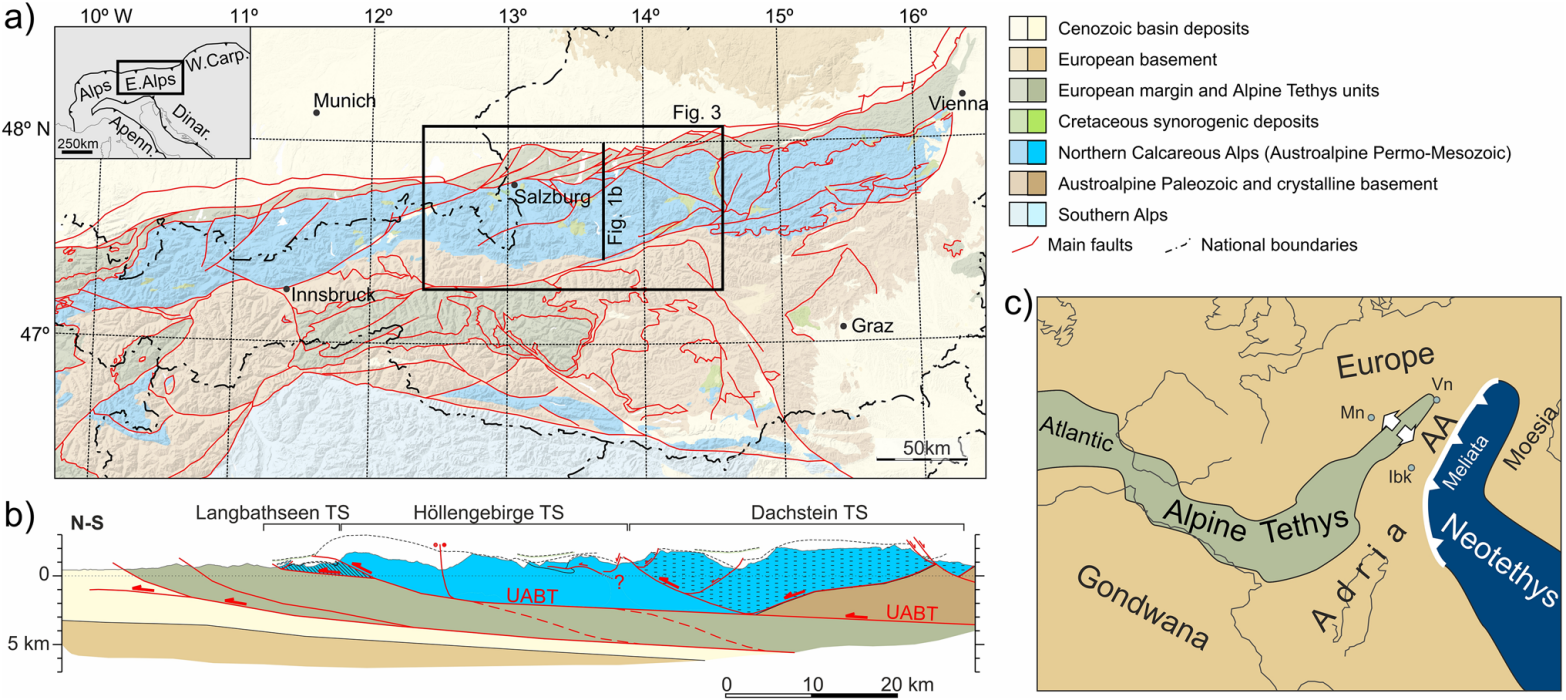
**a** Simplified geological map of the Eastern Alps (adapted from Schuster et al. 2015). **b** Synthetic geological cross-section across the central Northern Calcareous Alps. TS: thrust sheet; UABT: Upper Austroalpine basal thrust. **c** Paleogeographic reconstruction of the Austroalpine (AA) domain in relation to Europe and surrounding oceans at Late Jurassic times (adapted from Schuster et al. 2019). Ibk: Innsbruck, Mn: Munich, Vn: Vienna

The Eastern Alps comprise a stack of units from three tectonic provinces (Schmid et al. 2008) (Fig. 1c): Europe and its southern passive margin; the Alpine Tethys; and the Austroalpine domain (a segment of the Adria microcontinent). From Triassic to Middle Jurassic time, the Austroalpine domain was characterized by a salt tectonics-dominated passive margin that faced southeastwards into the Neotethys (or Meliata Tethys) ocean (Schmid et al. 2008; Schuster et al. 2019; Strauss et al. 2023) and, from Early to Late Jurassic times, by a passive margin that faced northwestward into the Alpine Tethys (or Penninic) ocean (Schmid et al. 2008; Schuster et al. 2019). Partly coeval with opening of the Alpine Tethys, subduction of the Meliata Tethys initiated in the Early Jurassic (Plašienka 2018) and culminated with obduction in the Late Jurassic (Schmid et al. 2008), leading to the first contractional phase in the Austroalpine domain. Subsequently, intra-continental collision and subduction occurred within the Austroalpine domain during the Cretaceous (Miladinova et al. 2022).

Whereas Cretaceous intra-continental subduction is well documented based on records of metamorphism (Miladinova et al. 2022), the precise nature of Late Jurassic tectonism is still debated. In the absence of evidence for metamorphism of this age, authors have based their interpretations on isolated observations of tectono-sedimentary relationships or petrological provenance analyses. Interpretations range from either transtension and/or transpression (Frank and Schlager 2006; Ortner et al. 2008; Mandl 2013; Ortner 2017), to subduction with associated thrusting (Gawlick et al. 1999; Frisch and Gawlick 2003), or to accretionary prism development (Gawlick and Missoni 2019).

During the main phase of Alpine orogenesis, lasting from Late Cretaceous to Early Miocene times, the Austroalpine domain was thrust over the Alpine Tethys. Subsequently, both tectonic units together overrode the European margin, whereby the proximal foreland succession became involved in thrusting (Faupl and Wagreich 2000; Schmid et al. 2008; Schuster et al. 2019). A final phase of deformation involved the exhumation of the axial zone of the Eastern Alps and the lateral extrusion along the orogen (Ratschbacher et al. 1991; Linzer et al. 2002).

### Stratigraphy

**Fig. 2:**
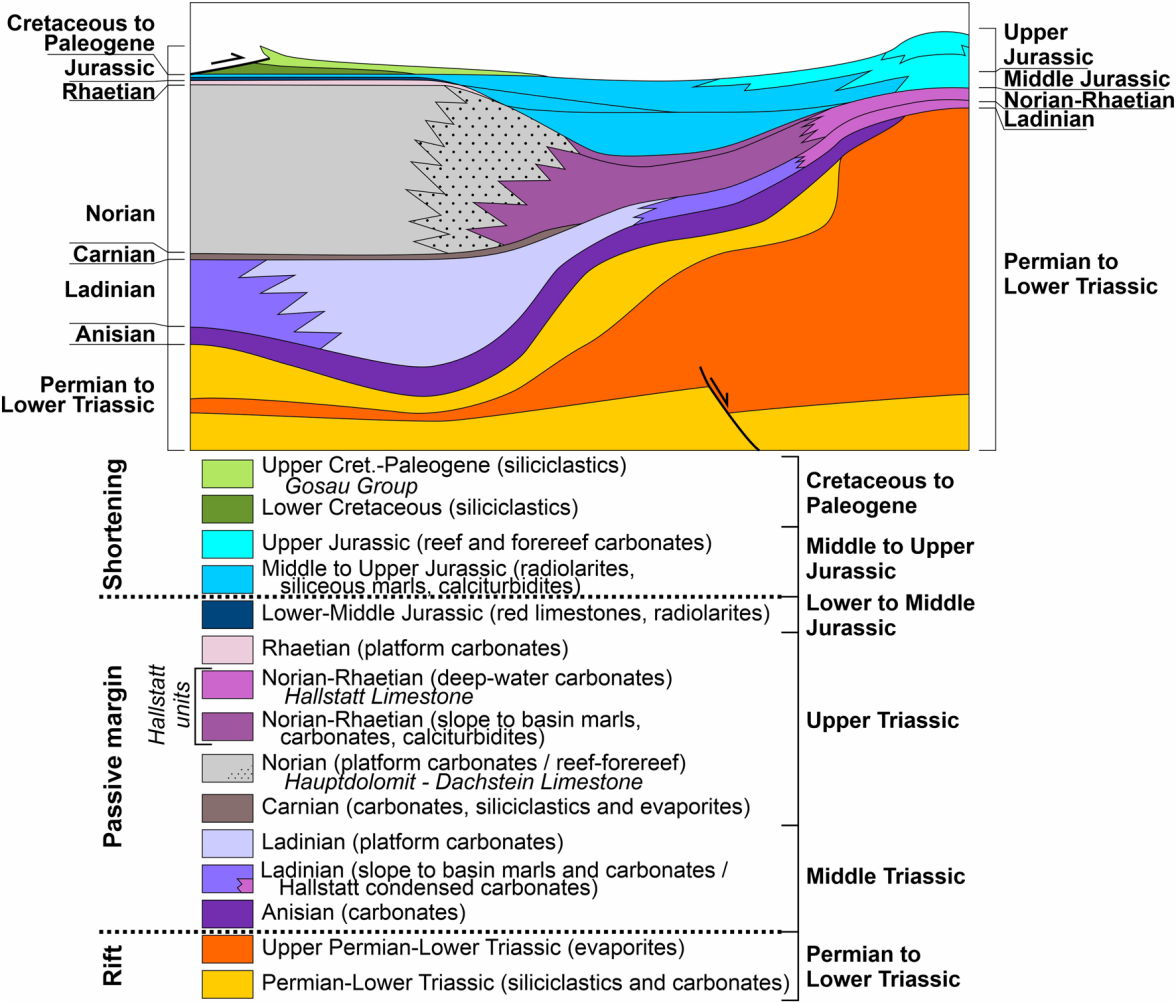
Synthetic chronostratigraphic chart of the Northern Calcareous Alps (adapted from Mandl 2000 and Fernandez et al. 2022)

The Permo-Mesozoic succession of the central NCA (see, e.g., Schlager and Schöllnberger 1974; Tollmann 1976a; Mandl 2000; Gawlick et al. 2009) (Fig. 2) was originally underlain by Paleozoic units that presently form most of the antiformal stack of the axial zone of the Eastern Alps. In the central NCA, basal successions of Permian red beds are overlain by an Upper Permian to Lower Triassic sequence of evaporites and shallow-marine clastics that ranges in thickness from a few hundreds to more than 1000 m thick. Above them lies an Anisian (Middle Triassic) succession of shallow- and deep-water limestones up to a few hundreds of meters; this succession is interpreted to represent the end of rifting (Leitner and Spötl 2017; Strauss et al. 2021). The Anisian carbonates, in turn, are overlain by thick (in the order of 1000 m) mostly Ladinian (Middle Triassic) shallow-water carbonate platforms comprising lagoons, reefs, and steep slopes. The Ladinian platforms locally interfinger with and prograde over a much thinner package of basinal shales to limestones. Although the Ladinian platform continued growing until the early Carnian, it is referred to in the text and figures simply as Middle Triassic platform. During the subsequent Carnian (Late Triassic) pluvial episodes (cf., Ogg 2015), the Ladinian platform-and-basin ensembles became buried with a cyclothemic, mixed siliciclastic-carbonatic-evaporitic succession of highly variable thickness (zero to a few hundreds of m) (e.g., Bechstädt and Schweizer 1991).

**Fig. 3:**
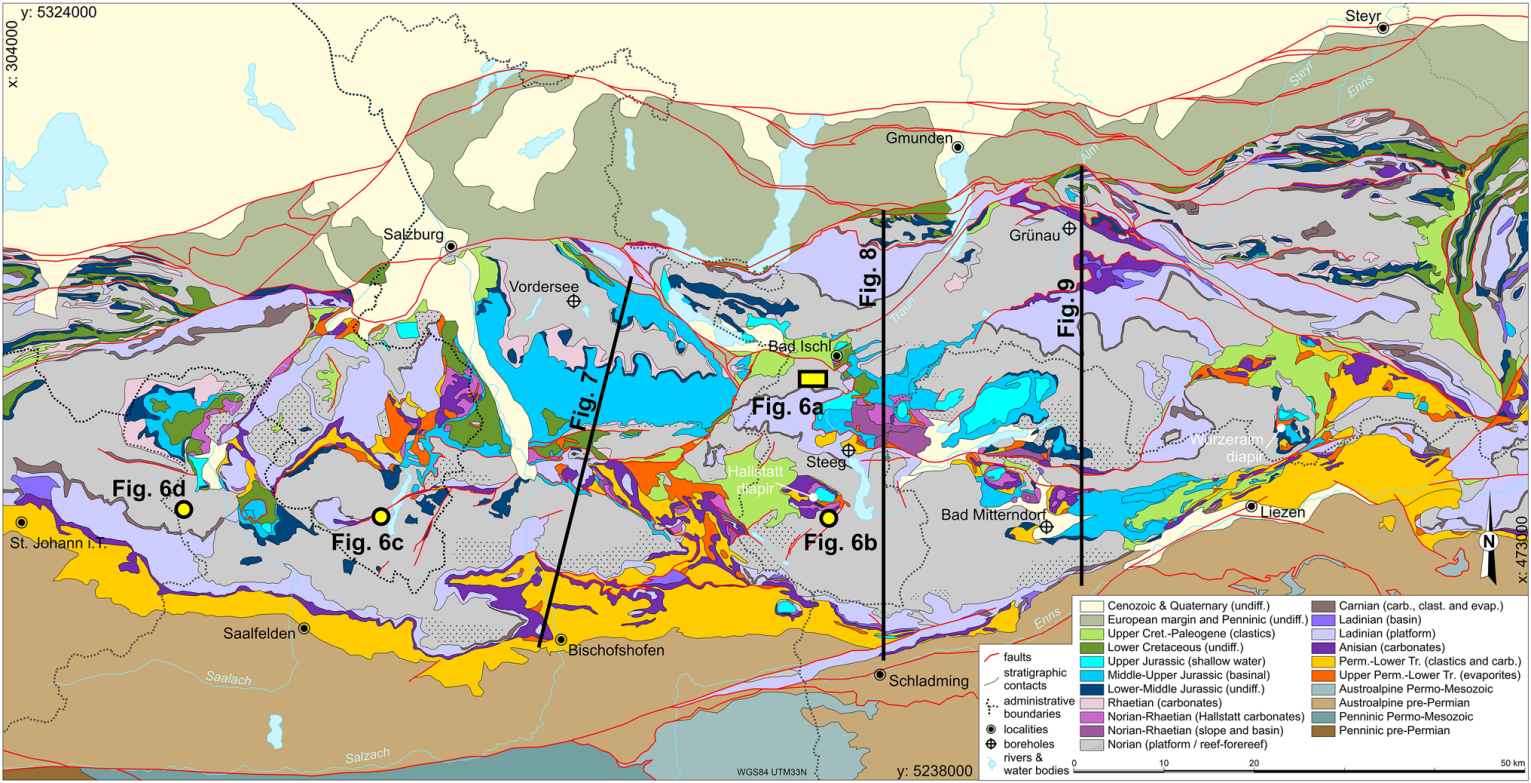
Geological map of the central Northern Calcareous Alps (adapted from Krenmayr 2005 and Krenmayr and Schnabel 2006)

Above the lower to middle Carnian succession, an extensive carbonate platform established that persisted from the late Carnian to the late Norian (Late Triassic). This platform, termed Hauptdolomit–Dachsteinkalk megabank, represents the most widespread carbonate rock unit of the NCA (e.g., Fig. 3), and locally attains ~ 2000 m in thickness. In the central NCA, the platform is represented by the—mostly undolomitized—Dachstein Limestone that comprises peritidal cyclic successions (“lagoonal Dachstein Limestone”), reefal to peri-reefal deposits (“reefal Dachstein Limestone”), and their slope to proximal basin equivalents. For simplicity, the Dachstein Limestone is termed in the text and figures Upper Triassic shallow-water carbonates. During the Rhaetian stage (Late Triassic), a single large Hauptdolomit–Dachsteinkalk megabank (cf., Mandl 2000) became differentiated into shallow basins with intercalated small carbonate shelves. Locally, in contrast, deposition of the Dachstein Limestone and its Rhaetian slope to basinal equivalent range up to the top of the Triassic.

Contemporaneous with the Upper and, locally, also with the Middle Triassic carbonate platforms, diverse types of (locally dolomitized) deep-water limestones accumulated in the so-called Hallstatt basins. These deep-water rocks are traditionally grouped under the informal term Hallstatt facies (or units). The archetypical Hallstatt facies (referred to here as the Hallstatt Limestone) is a stratigraphically condensed package of fossil-rich (particularly ammonites) flaser-bedded to stylonodular, red-colored limestone. The Hallstatt Limestone interfingers with allodapic carbonates shed from the platforms and their slopes that are grouped here with the Hallstatt Limestone under the term Hallstatt units. The original location of the Hallstatt basins relative to the time-equivalent Upper Triassic carbonate platforms is discussed below.

At the end of Triassic time, the Hauptdolomit–Dachsteinkalk megabank was tectonically segmented and drowned. From the Early to Middle Jurassic, a differentiated seafloor topography gave rise to deposition of packages of condensed, red shallow- to deep-water limestones (rosso ammonitico) on submarine swells, while thick successions of cherty deep-water limestones accumulated in the intercalated basins. During the terminal Middle Jurassic, probably mainly due to increased nutrient availability (Baumgartner 2013), sedimentation switched from calcareous to siliceous (radiolarites, siliceous marls); concomitantly, turbiditic deposition related to contractional tectonism started (Frisch and Gawlick 2003). Continued shortening throughout the Late Jurassic led to local shallowing over structural swells, followed by the establishment of isolated carbonate platform-to-slope systems.

The Triassic–Jurassic are covered, mostly unconformably and in separate basins, by Cretaceous to Paleogene synorogenic deposits that accumulated during thrusting: from Lower Cretaceous synorogenic clastics deposited during early imbrication of the NCA thrusts to Upper Cretaceous to Paleogene clastics (Gosau Group) that were deposited synchronous with decoupling of the NCA from their basement and northward thrusting over the Alpine Tethys (Faupl and Wagreich 2000). The stratigraphic record associated to Paleogene northward thrusting and Miocene lateral extrusion is absent (eroded) over most of the central NCA (Frisch et al. 1998; Ortner and Stingl 2001; Egger et al. 2017).

### Background on our understanding of the origin of the Hallstatt units

**Fig. 4:**
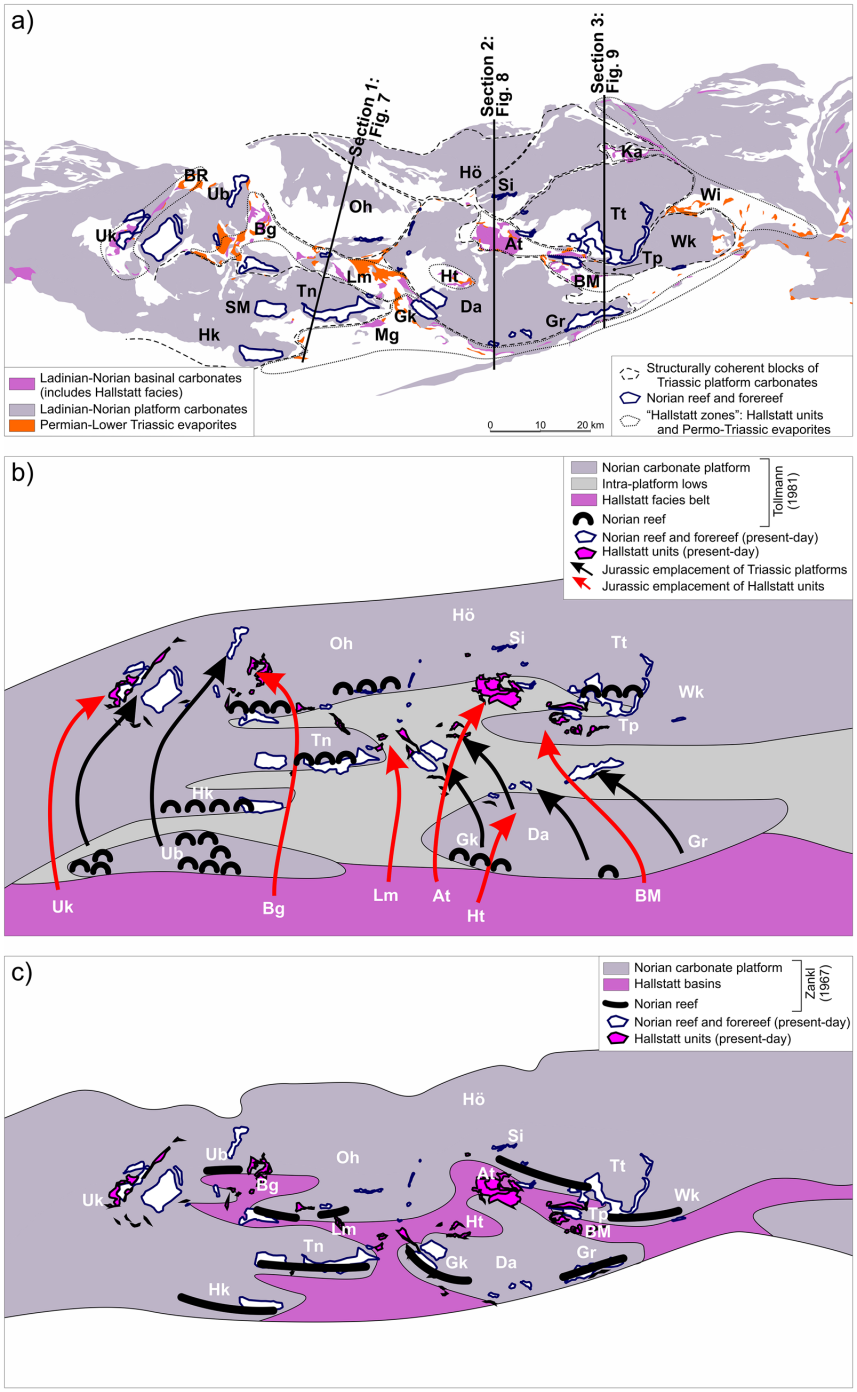
**a** Map simplified from Fig. 3, showing the present-day distribution of Middle to Upper Triassic shallow-water carbonates and Hallstatt units, and Permo-Triassic evaporites. Upper Triassic reefs have been mapped from published 1:50,000 scale geological maps (Plöchinger 1982, 1987; Schäffer 1982; Mandl and Matura 1995; Pavlik 2007, 2009, 2013a, b, 2014; Moser 2014) and correspond to either reef or near-reef facies within the otherwise lagoonal Upper Triassic platform. Hallstatt units crop out in elongated clusters marked by the dashed lines and named informally as follows: At: Altaussee; Bg: Berchtesgaden; BM: Bad Mitterndorf; BR: Bad Reichenhall; Gk: Gosaukamm; Ka: Kasberg; Lm: Lammertal; Mg: NCA southern margin; Uk: Unken; Wi: Windischgarsten. Blocks of platform domain bounded by the Hallstatt zones are labeled as: Da: Dachstein; Gr: Grimming; Hk: Hochkönig; Hö: Höllengebirge; Oh: Osterhorngruppe; Tn: Tennengebirge; Tp: Tauplitzalm; Tt: Totes Gebirge; Si: Singereben; SM: Steinernes Meer; Ub: Untersberg; Wk: Warscheneck. **b** Paleogeographic distribution of Upper Triassic reefs and representation of the southern Hallstatt domain relative to the northern Upper Triassic platform adapted from Tollmann (1981), with an overlay of present-day location of reefs and Hallstatt units. Arrows indicate approximate transport of allochthonous units and connect equivalent features in their Triassic and present-day locations. **c** Paleogeographic distribution of Upper Triassic reefs and representation of the Hallstatt basins adapted from Zankl (1967) with an overlay of present-day location of reefs and Hallstatt units

Upper Triassic Hallstatt units crop out in the central NCA in domains otherwise dominated by Upper Triassic shallow-water platform carbonates (lagoonal and reefal Dachstein limestone) (Fig. 3). The Hallstatt units typically cluster along specific elongate regions that trend roughly NW–SE and NE–SW (Fig. 4a). These regions (referred to traditionally as Hallstatt “zones”) are further characterized by abundant Permo-Triassic evaporites (Fig. 4a) and Lower Triassic clastics. Three of these regions are the subject of this contribution: the Lammertal Zone, the Altaussee Zone, and the Bad Mitterndorf Zone (“Lm,” “At,” “BM” in Fig. 4a).

The proximity of Hallstatt units and Permo-Triassic evaporites in outcrop and the evidence for syndepositional fracturing of the Hallstatt units (Schlager 1969) has led authors to interpret that the Hallstatt units were deposited in a deep-water domain above growing diapirs (e.g., Mandl 2000; Schorn and Neubauer 2014; Gawlick and Missoni 2019). It is consistently observed that the Hallstatt units and accompanying evaporites and clastics rest on Middle to Upper Jurassic strata that in turn blanket the Upper Triassic platform units (Mandl et al. 2012; Mandl 2013; Gawlick and Missoni 2015, 2019; Fernandez et al. 2021). Locally, along with Hallstatt units, kilometer-sized blocks of Triassic platform carbonates also rest structurally on Jurassic beds and their underlying Triassic platforms (i.e., a duplication of Triassic platforms; Braun 1998; Gawlick and Missoni 2019; Ortner et al. 2022). Likewise, Permo-Triassic evaporite masses and Lower Triassic clastics, without associated Hallstatt units, can rest on Jurassic rocks (Mandl et al. 2012; Fernandez et al. 2021; Kurz et al. 2023).

As previously discussed, it is commonly accepted that the Hallstatt units originated in a distal (southward) position of the NCA margin and were then transported in the Late Jurassic over large distances (tens of kilometers) onto the Upper Triassic platform carbonates (Fig. 4b). Different models have been put forward for the northward transport of the Hallstatt units: (1) the *Gleittektonik* model (Tollmann 1981; who was inspired by the work of Plöchinger and Draxler 1974) postulates that Late Jurassic uplift of the distal margin led to the northward gravitational sliding of large fragments (olistoliths) of the Hallstatt units (and evaporites) into their present-day positions; (2) the accretionary prism model interprets the Hallstatt and accompanying units as part of a Jurassic accretionary prism, of which only fragments are preserved today (e.g., Gawlick et al. 1999; Gawlick and Missoni 2019); and (3) the thrust model interprets the Hallstatt unit outcrops as fragments of a large, relatively thin thrust sheet originated from the distal margin (Strauss et al. 2023).

Prior to the current acceptance of an allochthonous origin for the Hallstatt units, some authors defended the possibility that these units actually deposited within intra-platform seaways (known as Hallstatt “channels”) (Zankl 1967; Schlager 1967; Häusler 1980) (Fig. 4c). The main objection to this model is the general absence of fully preserved platform-to-basin transitions that would be expected for an intra-platform origin of the Hallstatt units. One such transition from Upper Triassic platforms into Hallstatt units occurs along the southern margin of the NCA, in the Gosaukamm ("Gk" in Fig. 4a) (Kenter and Schlager 2009). This transition is, however, generally accepted to be a transition into the broad Hallstatt domain that lay south of the Triassic platforms (Fig. 4b). Other possible transitions documented by Häusler (1980), in the Lammertal, and Häusler and Berg (1980), in Unken (see "Lm" and "Uk" in Fig. 4a for approximate locations), have mostly been sidelined by posterior authors due to the strong tectonic overprint in both areas (assuming it implies allochthony) and the Rhaetian age of some of the elements in the Unken area (as opposed to a predominantly Norian age of the Hallstatt units).

## Structural configuration of the central NCA

**Fig. 5:**
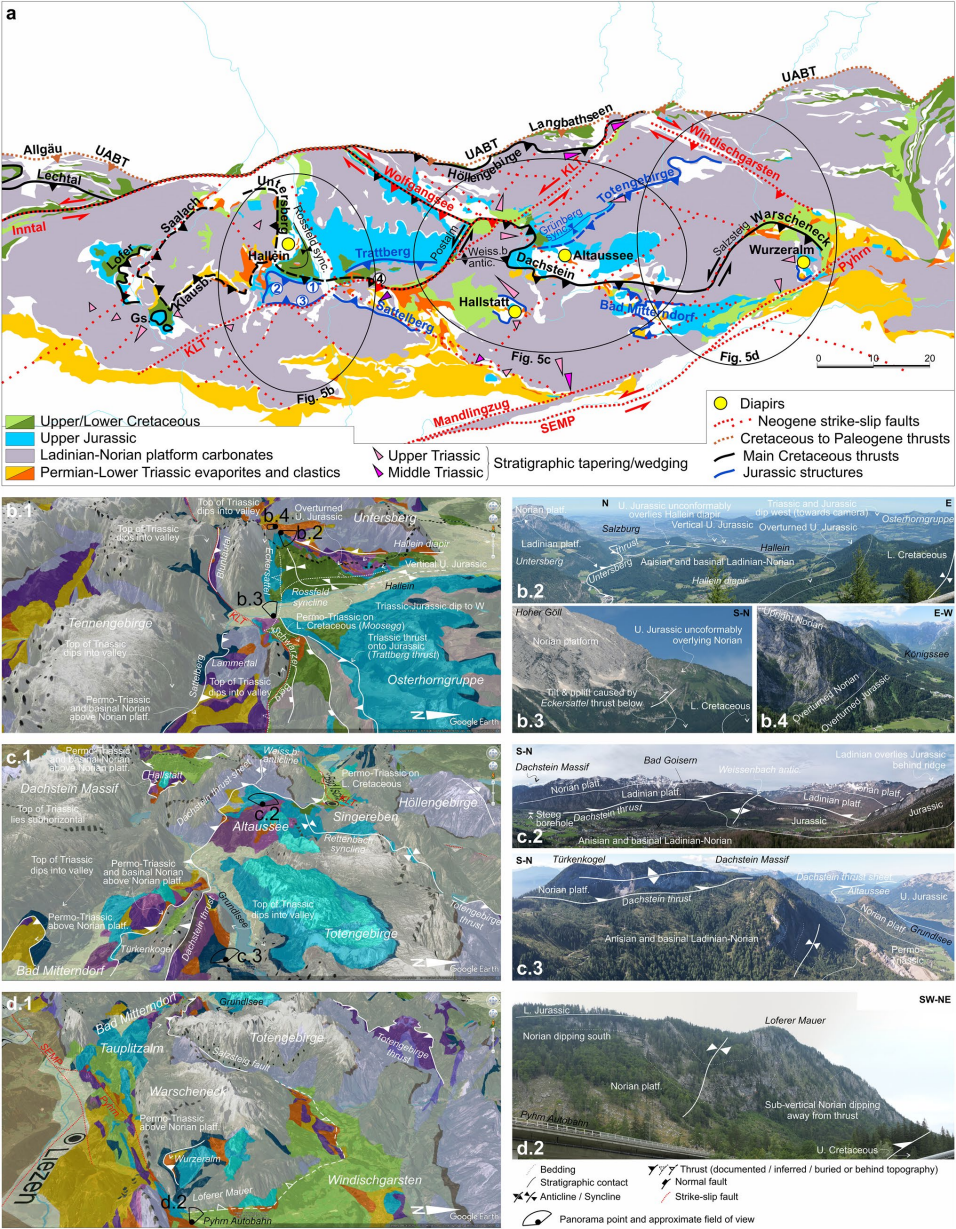
**a** Simplified structural framework of the central NCA (area equivalent to that in Fig. 3) including localities with structures related to Triassic salt tectonics (growth wedges, diapirs) and regional dips. Structures drawn, and partly simplified, from Tollmann 1976b, Braun 1998, Linzer et al. 1995, Ortner and Kilian 2022. UABT: Upper Austroalpine basal thrust; Gs.: Gerhardstein; Klausb.: Klausbach; KLT: Königsee–Lammertal–Traunsee fault system; SEMP: Salzach–Enns–Mariazell–Puchberg fault system; Weiss.b.: Weissenbach; sync.: syncline; antic.: anticline. Structure numbers: 1: Eckersattel; 2: Dürreckberg; 3: Bluntautal; 4: Schwarzer Berg. It is noted that the UABT locally coincides with the Langbathseen, Höllengebirge, and Allgäu thrusts. See text for details. **b**–**d** Details on the interpretation of the eastern, central and western segments of the study area. **b.1**, **c.1**, and **d.1** show oblique aerial views, with north to the right on the 3D topography of GoogleEarth (2 × vertical exaggeration). **b.2, b.3, b.4, c.2, c.3, d.2** show panorama photographs of key structures in the area (see text for details). The location of the panorama photographs is shown in **b.1**, **c.1** and **d.1**

Within the Eastern Alps, the Northern Calcareous Alps (NCA) are an Upper Permian to Paleogene sedimentary succession, part of the Upper Austroalpine unit (Schmid et al. 2004), that has been almost entirely decoupled from its pre-Permian basement. The NCA Permo-Mesozoic lies at present directly above imbricated Alpine Tethys and European units, and, along its trailing edge, above its pre-Permian basement (Fig. 1a, b). The NCA have traditionally been sub-divided into three major thrust systems; from lower to higher and from foreland to hinterland these are the Bajuvaric, the Tirolic, and the Juvavic thrust systems (Hahn 1913a, b; Mandl 2000) (equivalent in the central NCA to the Langbathseen, Höllengebirge, and Dachstein thrust sheets; Tollmann 1976b; Figs. 1b, 5, Table 1). Imbrication of these units occurred in the Early to Late Cretaceous (Mandl 2000; Ortner and Kilian 2022; Levi 2023), and from the Late Cretaceous they were further thrust over units of the Alpine Tethys and southern European margin (Faupl and Wagreich 2000). At present, the NCA are floored by the UABT (Upper Austroalpine basal thrust) (Fig. 5).

**Table 1:** List of key structural elements of the central NCA (as illustrated on Fig. 5) with their inferred kinematics, age, and criteria for these interpretations

Structure	Figure	Type	Age	Criterion/a	Transport direction (top to, vergence)	Criterion/a	Shortening/Displacement	Criterion/a
Totengebirge thrust	5a, 5c.1, 5d.1	Thrust	Late Jurassic	No strata younger than Norian in the flat-on-flat contact at Kasberg (Egger and van Husen 2007) and along-strike prolongation into the Grünberg syncline (Grünberg Mulde of Tollmann 1976b)	NW	Striations at Kasberg (Linzer et al. 1995)	> 8 km	Restoration (Linzer et al. 1995)
Grünberg syncline	5a, 5c.1	Syncline	Late Jurassic	Growth geometry in syncline capped by Upper Jurassic (Missoni and Gawlick 2011; Mandl et al. 2012; Mandl 2013)	NW	SW–NE trend of syncline	< 5 km	Restoration (this paper)
Trattberg thrust	5a, 5b.1	Thrust	Late Jurassic	Growth geometry in Upper Jurassic slope deposits (Ortner 2017)	N	Fault kinematic indicators	< 5 km	Restoration (this paper)
		Normal	post-Lower Cretaceous	Part of a system of normal faults, with Lower Cretaceous as the youngest strata in the hanging wall	S	Fault kinematic indicators	< 5 km	Restoration (this paper)
Eckersattel thrust	5a, 5b.1, 5b.3	Thrust	Late Jurassic	Erosional unconformity of hanging wall and onlap by Upper Jurassic interpreted to relate to uplift (equivalent to that on Trattberg)	N to NE?	E–W to NW trending folds^a^ in hanging wall and footwall (Braun 1998)	< 5 km	Unconstrained, by analogy to Trattberg
		Thrust	post-Lower Cretaceous	Folding of Lower Cretaceous north of the Hoher Göll	N to NE?	E–W trend of southern Rossfeld syncline^a^	< 2 km	Limited folding in Rossfeld syncline
Dürreckberg thrust	5a, 5b.4	Thrust	Late Jurassic	Youngest strata in footwall are Upper Jurassic slope deposits	Uncertain		Uncertain	
Sattelberg thrust	5a, 5b.1	Thrust	Late Jurassic	Youngest strata in footwall are Upper Jurassic basin deposits	S	Outcrop-scale structures (Cornelius and Plöchinger 1952; Schweigl and Neubauer 1997)	> 2 km	N–S dimension of outcropping hanging wall (in flat-on-flat situation) is the minimum displacement magnitude
Bluntautal thrust	5a, 5b.1	Thrust	Late Jurassic	By analogy to Sattelberg thrust along strike	S	By analogy to Sattelberg thrust along strike	> 4 km	Hanging wall Triassic rests on top of the footwall top of Triassic. A 30° dipping ramp needs to be 4 km to raise the hanging wall 3 km (estimated thickness of the entire Triassic stratigraphy in the footwall)
Hallstatt south and north	5a, 5c.1	Salt allochthon	Late Jurassic	Based on Fernandez et al. (2021) and by analogy to Wurzeralm	S and N	Salt stock is known to stretch downwards under the Hallstatt salt mine (Fernandez et al. 2021)	> 2 km	Restoration (Fernandez et al. 2021)
Bad Mitterndorf south and north	5a, 5c.1	Salt allochthon	Late Jurassic	By analogy to Hallstatt and Wurzeralm	S and N	By analogy to Hallstatt	Uncertain	
Wurzeralm	5a, 5d.1	Salt allochthon	Late Jurassic	Based on Kurz et al. (2023)	S	Shear fabric in hanging wall (Kurz et al. 2023)	> 2 km	Cross-section (Kurz et al. 2023)
Dachstein thrust	5a, 5c.1, 5c.2, 5c.3	Thrust	Early Cretaceous	Youngest strata in footwall are Lower Cretaceous (Mandl et al. 2012; Levi 2023) and Dachstein thrust buried by Gosau strata (Levi et al. 2022)	Uncertain: NW, N or NE	NW directed could be inferred from analogy to contemporaneous western NCA thrusts (Linzer et al. 1995; Kilian and Ortner 2019; Ortner and Kilian 2022) and Late Cretaceous shear sense in Austroalpine metamorphic basement units (Ratschbacher and Neubauer 1989)	> 30 km if NW directed > 10 km if N directed < 10 km if NE directed	For all scenarios it is understood that the main thrust displacement occurred in the Early Cretaceous. The hanging wall of the Dachstein thrust rests on a footwall flat of Upper Jurassic rocks that is at roughly 5 km in a N–S direction (Fig. 5c.2; Fernandez et al. 2022; Levi 2023). The Steeg well (Figs. 3, 5c.2; Mandl et al. 2012) indicates the footwall ramp in the Triassic must lie to its south
				Clasts of Hallstatt Limestone contained in footwall Lower Cretaceous strata (Krische and Gawlick 2015)		N directed would be based on E–W trending folds in the hanging wall and footwall (Levi 2023) and E–W trend of Lawinenstein–Türkenkogel anticline (Tollmann 1960)		If thrusting is NW directed, the Dachstein thrust sheet must come from SE of the Altaussee area
						NE directed would be based on analogy to Untersberg thrust and to NE-directed thrusting the Schwarzer Berg on Lower Cretaceous (Decker et al. 1994) and would contemplate the Postalm fault as a lateral ramp		If thrusting is N directed, the Dachstein thrust sheet must come from S of the Steeg well
								If thrusting is NE directed, the Dachstein thrust sheet comes out-of-the-plane in panorama in Fig. 5c.2 and restores SE of the Upper Jurassic footwall flat
		Thrust	Post-Cretaceous	Folded and tilted Gosau strata (Levi 2023)	N to NE	Based on fold axes in folded^a^ Gosau strata (Levi 2023)	< 5 km	No evidence for major uplift or displacement of Gosau units (Levi 2023)
Untersberg thrust	5a, 5b.1, 5b.2	Thrust	Early Cretaceous	Youngest strata in footwall to the east are Lower Cretaceous (Krenmayr et al. 2009) Thrust sealed along its northern termination by Gosau strata (Schweigl and Neubauer 1997)	NNE–NE	NNE- to NE-directed fault slickensides and Pre-Gosau NNE to NE directed shortening recorded by folding and faulting in the footwall of the Untersberg thrust (Schweigl and Neubauer 1997)	> 1 km	Hanging wall Triassic rests on top of the footwall top of Triassic. A 30° dipping ramp needs to be over 500 m to raise the hanging wall 500 m (estimated thickness of the condensed Triassic stratigraphy in the footwall)
Rossfeld syncline (northern segment)	5a, 5b.1	Syncline	Early to Late Cretaceous?	Early Cretaceous by analogy to overthrust Untersberg thrust, but in general unconstrained end of growth. Youngest strata involved in syncline are Aptian in age (Schweigl and Neubauer 1997)	NNE–NE?	Folding-related flexural slip slickensides indicate NNE to NE vergence (Schweigl and Neubauer 1997) Synclinal axis trends N–S to the east of the Hallein diapir and WSW–ENE to the south of the diapir—axis rotation likely relates to influence of the diapir	> 1 km	Minimum length of overturned limb (Schweigl and Neubauer 1997)
Lofer-Gerhardstein thrusts	5a	Thrust	Early Cretaceous	Age of youngest strata in thrust footwall (Pavlik 2007)	N and NW	Shear fabrics indicating top to N in footwall of Gerhardstein thrust and NW shortening in Lofer (Rittner 2006)	Uncertain	Uncertain restored location of thrust sheets
Klausbach thrust	5a	Backthrust	Early Cretaceous	Age of youngest strata in thrust footwall (Pavlik 2007)	SE?	Thrust shows flat-on-flat geometry, with no truncation of footwall or hanging wall units, which is interpreted to mean its map expression is a strike section (normal to transport). Se-directed transport is inferred based on E-directed Untersberg thrust (which lies along strike and is of same age)	> 4 km	Hanging wall Triassic rests on top of the footwall top of Triassic. A 30° dipping ramp needs to be 4 km to raise the hanging wall 3 km (estimated thickness of the entire Triassic stratigraphy in the footwall)
Saalach thrust	5a	Thrust	Early Cretaceous	By analogy to Klausbach and Lofer thrusts	NW to NNW	NW- to NNW-directed shortening (Rittner 2006)	> 4 km	Hanging wall Triassic rests at least locally on top of the footwall top of Triassic. A 30° dipping ramp needs to be 4 km to raise the hanging wall 3 km (estimated thickness of the entire Triassic stratigraphy in the footwall)
Schwarzer Berg thrust	5a, 5b.1	Thrust	Early Cretaceous?	Lower Cretaceous basin limited to south by Schwarzer Berg unit	Uncertain		Uncertain	
		Thrust	Post-Cretaceous	Thrusting of Schwarzer Berg above Lower Cretaceous (Decker et al. 1994)	NE	Fault kinematic indicators (Decker et al. 1994)	Uncertain	
Permo-Triassic of Moosegg	5b.1	Allochthonous salt	Early Cretaceous	Permo-Triassic evaporites underlain (Plöchinger 1987) and overlain (proven by quarry excavation) by Lower Cretaceous strata	W	Shear fabric in Permo-Triassic gypsum (Schorn and Neubauer 2011)	Uncertain	
Permo-Triassic of Bad Ischl	5c.1	Allochthonous salt	Early Cretaceous	Permo-Triassic evaporites underlain and overlain by Lower Cretaceous strata	W	Shear fabric in Permo-Triassic gypsum (unpublished data)	Uncertain	
Höllengebirge thrust	5a	Thrust	Late Cretaceous	Youngest strata in footwall are Upper Cretaceous (Egger 1996)	N	Deformation in hanging wall consistent with N-directed shortening and transport (Levi 2023)	> 4 km	Restoration (this paper)
Langbathseen thrust	5a	Thrust	Late Cretaceous	Youngest strata in hanging wall are Upper Cretaceous (Egger 1996) and by analogy to Allgäu thrust	N	E–W trending folds^a^ in hanging wall (Egger 1996)	Uncertain	Cretaceous shortening unconstrained (excludes displacement on same thrust as UABT)
Lechtal thrust	5a	Thrust	Early Cretaceous	Ortner and Kilian (2022)				
Allgäu thrust	5a	Thrust	Late Cretaceous	Ortner and Kilian (2022)				
UABT	5a	Thrust	Late Cretaceous to Paleogene	Schweigl and Neubauer (1997); Faupl and Wagreich (2000)			> 50 km	Restoration (this paper and Linzer et al. 1995)
Weissenbach anticline	5a, 5c.1	Anticline	Post-Early Cretaceous	Folds the Dachstein thrust (Fernandez et al. 2022; Levi 2023)	N	E–W trending fold^a^ axis (Levi 2023)	~ 1 km	Restoration (Levi 2023)
Wolfgangsee	5a	Thrust/strike-slip fault	Eocene to Miocene	Age of Cenozoic rocks involved in faulting and cross-cutting relationships of kinematic indicators (Peresson and Decker 1997)	Dextral to sinistral transpression	Fault kinematic indicators (Linzer et al. 2002)	10–15 km	Based on distance required for uplift of sub-UABT units in oblique strike-slip (Linzer et al. 1995)
Windischgarsten	5a	Thrust/strike-slip fault	Eocene to Miocene	Age of Cenozoic rocks involved in faulting and cross-cutting relationships of kinematic indicators (Peresson and Decker 1997)	Dextral to sinistral transpression	Fault kinematic indicators (Linzer et al. 2002)	22 km	Based on offset of Gosau strata
KLT-Königsee segment	5a	Salt ridge	Triassic	Growth wedge in Upper Triassic Limestones (Fig. 6c)	Salt ridge	Likely elongate shape body precursor to KLT strike-slip fault	1–2 km?	Estimated shortening during welding
		Thrust, strike-slip fault	Oligocene to Miocene	Age of Cenozoic rocks involved in faulting and cross-cutting relationships of kinematic indicators (Peresson and Decker 1997)	Sinistral	Fault kinematic indicators (Decker et al. 1994). Outcrop of Middle Triassic limestones along fault (Decker et al. 1994) may have been facilitated by stratigraphic condensation	10–15 km	Based on map offset of base of NCA along lineaments interpreted from remote sensing images (Decker et al. 1994). It is noted that the base of the NCA dips roughly 30° and 10–15 km of horizontal offset in map view can be accounted for by 5–8 km of oblique offset
KLT-Bluntautal segment	5a, 5b.1	Thrust to strike-slip fault	Oligocene to Miocene	Age of Cenozoic rocks involved in faulting and cross-cutting relationships of kinematic indicators (Peresson and Decker 1997)	Sinistral	Fault kinematic indicators (Decker et al. 1994)	Undefined	
KLT-Lammertal segment	5a, 5b.1	Thrust to strike-slip fault	Oligocene to Miocene	Age of Cenozoic rocks involved in faulting and cross-cutting relationships of kinematic indicators (Peresson and Decker 1997)	Sinistral	Fault kinematic indicators (Decker et al. 1994)	8 km	Based on geometric model (Linzer et al. 2002)
KLT-Postalm segment	5a	Salt ridge?	Triassic?	Consistent outcrop of Permo-Triassic evaporites along fault without the presence of other Triassic units speaks for absent stratigraphy that might be expected above a salt structure	Salt ridge	Likely elongate shape body precursor to KLT strike-slip fault	1–2 km?	Estimated shortening during welding
		Thrust to strike-slip fault	Oligocene to Miocene	Age of Cenozoic rocks involved in faulting and cross-cutting relationships of kinematic indicators (Peresson and Decker 1997)	Sinistral	Fault kinematic indicators (Decker et al. 1994)	3 km	Offset of Wolfgangsee structures (Decker et al. 1994). Note that the Postalm segment of the KLT is also a boundary to the Cretaceous Dachstein thrust sheet, most likely indicating re-activation of a pre-existing structure
KLT-Bad Ischl segment	5a, 5c.1	Thrust to strike-slip fault	Oligocene to Miocene	Age of Cenozoic rocks involved in faulting and cross-cutting relationships of kinematic indicators (Peresson and Decker 1997)	Sinistral	Fault kinematic indicators (Decker et al. 1994; Levi et al. 2022)	Undefined	No markers (Levi et al. 2022)
KLT-Trauntal segment	5a, 5c.1	Thrust to strike-slip fault	Oligocene to Miocene	Age of Cenozoic rocks involved in faulting and cross-cutting relationships of kinematic indicators (Peresson and Decker 1997)	Sinistral	Fault kinematic indicators (Decker et al. 1994)	3 km	Offset of frontal NCA thrust (UABT) and Langbathseen thrusts (Egger 2007)
Mandlingzug fault	5a	Thrust to strike-slip fault	Oligocene to Miocene	Age of Cenozoic rocks involved in faulting and cross-cutting relationships of kinematic indicators (Peresson and Decker 1997)	Sinistral to transtensional	Fault kinematic indicators (Linzer et al. 1995, 2002)	undefined	
Pyhrn fault	5a, 5c.1	Strike-slip fault	Oligocene to Miocene	Age of Cenozoic rocks involved in faulting and cross-cutting relationships of kinematic indicators (Peresson and Decker 1997)	Sinistral to transtensional	Offset of interpreted equivalent features (Linzer et al. 2002)	28 km?	Offset of interpreted front of Dachstein thrust sheet (Linzer et al. 2002)
SEMP fault	5a, 5c.1	Strike-slip fault	Oligocene to Miocene	Age of Cenozoic rocks involved in faulting and cross-cutting relationships of kinematic indicators (Peresson and Decker 1997)	Sinistral to transtensional	Fault kinematic indicators (Linzer et al. 1995, 2002)	60 km?	Offset of Austroalpine basement units (Linzer et al. 2002) (assumes pure strike-slip, no dip-slip component)

^a^It I noted that fold trends indicate local shortening direction but can be unreliable indicators of tectonic transport direction, in particular in salt-rich settings (e.g., Duffy et al. 2018)

The fold-and-thrust structure of the central NCA was partly overprinted by NE–SW and NW–SE trending strike-slip corridors during the Neogene (Decker et al. 1994; Linzer et al. 1995, 2002) (Fig. 5). These faults are interpreted to detach on the UABT and do not exceed 10 km of strike-slip offset *within* the NCA (Linzer et al. 2002) (Table 1). These faults are consistently associated to outcrops of Permo-Triassic evaporites (Fig. 5; Levi et al. 2022) and to the main outcrops of Hallstatt units (Frisch and Gawlick 2003; cf. Figures 3, 4a, 5a).

A summary of the key structural elements of the study area is shown in Fig. 5 and listed with details on their interpreted kinematics and ages in Table 1. Some of these structural elements coincide with regions of outcrop of Hallstatt facies (“Hallstatt zones”; cf. Figure 4a). These structures and “Hallstatt zones” also bound a series of blocks that have little internal deformation and are tens of kilometers across (Fig. 4a) and are dominated by Triassic platform carbonates 2–4 km in thickness. The relationship between the “Hallstatt zones” and these platform blocks is the target of the cross-sections in this study. Of particular relevance here are the Tennengebirge and Osterhorngruppe blocks (Section 1), the Dachstein, Singereben, and Höllengebirge blocks (Section 2), and the Grimming, Tauplitzalm, and Totengebirge blocks (Section 3).

### Salt tectonics

**Fig. 6:**
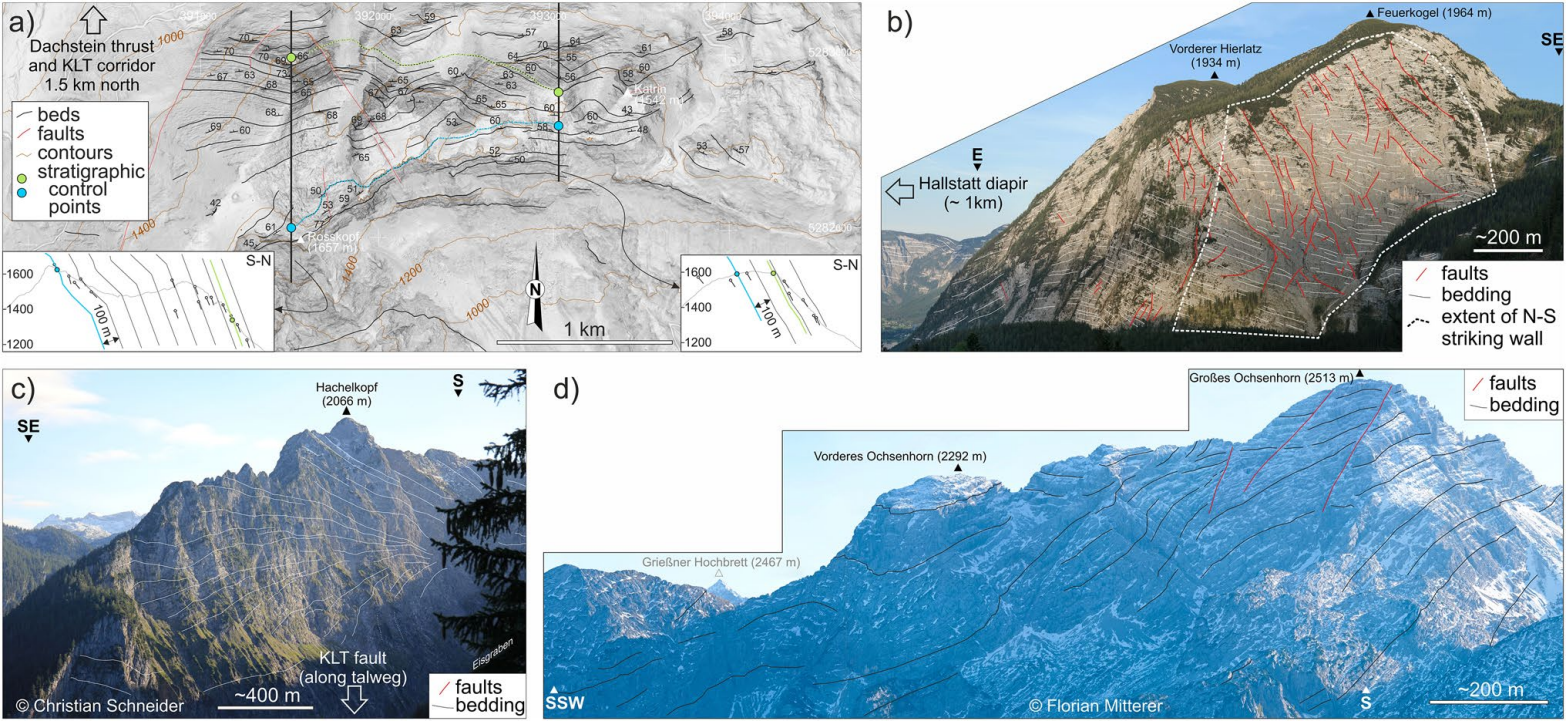
Examples of stratigraphic thinning of the Dachstein Limestone (see Fig. 3 for location) **a** Map of Dachstein Limestone bedding traces in the Mount Katrin area (near the Dachstein thrust and the KLT fault, see Fig. 3 for location). The cross-sections in the insets show thinning between the green and blue marker beds, from over 700 m in the west to under 200 m in the east. The background image is a hillshade of airborne laserscan topography (0.5 m resolution; source: Land Oberösterreich, data.ooe.gv.at). **b** Photo of the Hierlatzwand showing stratigraphic thinning toward the Hallstatt diapir (see Fig. 3 for location). **c** Sedimentary growth wedge on the Hachelwand, along the southwestern end of the KLT fault (See Fig. 3 for location). **d** Panorama of the Großes Ochsenhorn in the Loferer Steinberge (see Fig. 3 for location) with SW-directed tapering of beds. **c** and **d** Reproduced with permission of the authors

The evolution of the NCA has been strongly conditioned by salt tectonics since Triassic times (Granado et al. 2019; Fernandez et al. 2021, 2022; Strauss et al. 2021, 2023; Santolaria et al. 2022; Kurz et al. 2023). In its simplest form, salt tectonics (see Table 2 for a list of key terms used here) has been recorded by the development of growth wedges in shallow-water platform carbonates (Figs. 5, 6). These wedges indicate that the base of the Triassic succession subsided differentially, while the depositional surface stayed near-horizontal and at neritic depths (as discussed by Strauss et al. 2021). The rapid lateral thickness changes observed and extreme rotation of some beds (over 50° in Fig. 6c) are best explained with a shallow detachment at a depth compatible with the underlying Permo-Triassic evaporites. Halokinesis was likely triggered and driven by Triassic crustal extension, first related to Meliata Tethys rifting and downslope gliding (raft tectonics) (Strauss et al. 2023) and subsequently to Alpine Tethys rifting (Bertotti et al. 1993; Héja et al. 2018). Many of the Triassic growth wedges occur along or near structures that recorded post-Triassic activity (Figs. 5a, 6a, c), indicating that re-activation of Triassic-age salt structures likely played an important role in the post-Triassic structural configuration of the NCA. A similar history of re-activation of Triassic-age salt structures has been documented by Oravecz et al. (2023), for the Western Carpathian Silica Nappes (a province with an evolution that mirrors that of the NCA, Mandl 2000).

**Table 2:** Glossary of salt tectonics terms, adapted from Jackson and Hudec (2017), Rowan (2017) and Rowan and Giles (2021)

Term	Definition
Halokinesis	Salt tectonics style in which salt motion is driven predominantly by gravitation (e.g., due to overburden, downslope gliding) with limited tectonic input
Diapir	Salt accumulation that is discordant with its overburden. Typically salt in diapirs has an anomalous stratigraphic position. Diapirs are often stock-shaped structures
Salt stock	Sub-vertical diapiric structure that is roughly cylindrical in shape
Salt wall	Diapiric structure with elongate shape in map view (forming ridge-like structures)
Salt weld	Surface across which strata are juxtaposed that were originally separated by salt
Roof	Strata accumulated above a diapir, normally presenting a condensed stratigraphy
Minibasin	Depocenters developed by subsidence into underlying salt that are adjacent to one or more diapirs, salt walls or equivalent salt structures
Raft tectonics	Extreme form of halokinesis in which downslope gliding leads to extreme extension, with the formation of isolated minibasins (rafts) separated by through-going salt structures (salt walls or equivalent)
Autochthonous salt	Salt that is in its original stratigraphic position
Allochthonous salt	Sheetlike and near-horizontal (at low angles to strata) salt bodies emplaced above their original stratigraphic position. Most allochthons form by extrusion from an original salt body to near or onto the seabed

Furthermore, other potentially halokinetically related geometries have been described in the central NCA, for instance,

1. Tollmann (1960), Schöllnberger (1973), and Schmid et al (2003) document stratigraphic thinning of the Upper Triassic platform carbonates in the area of the Türkenkogel–Tauplitzalm (Fig. 5c.3);
2. Fernandez et al. (2022) documented onlap (and thinning) of Upper Triassic carbonates onto underlying Middle Triassic carbonates in the hanging wall of the Dachstein thrust sheet, and Neubauer and Genser (2018) discuss the lateral pinchout of the Middle Triassic platforms in the Tennengebirge;
3. Cornelius and Plöchinger (1952) and Häusler (1980) documented a major change in the thickness (from ~ 150 m to around 800 m in under one kilometer distance) of the Anisian carbonates in the vicinity to Section 1.

Of particular relevance to the study area is the relationship between the Hallstatt units and salt tectonics. The commonly accepted model of an allochthonous origin of the Hallstatt units implies that accompanying Permo-Triassic evaporites are also allochthonous. However, Fernandez et al. (2021, 2022) and Kurz et al. (2023) have shown that the Hallstatt and Wurzeralm diapirs (Figs. 3, 5) grew within the Upper Triassic Dachstein platforms (i.e., surrounded by shallow-water lagoonal environments). This is consistent with observations by Medwenitsch and Schauberger (1951) documenting Permo-Triassic evaporites under the Upper Triassic Dachstein Limestone in the Hallstatt salt mine. The Hallstatt diapir is overlain by, and has incorporated blocks of Hallstatt units (Schauberger 1955; Mandl et al. 2012; Schorn and Neubauer 2014; Fernandez et al. 2021) whose presumed allochthonous origin is at odds with the parautochthonous origin of the Permo-Triassic evaporites.

With this in mind, the aim of this article is to further explore the implications of interpreting the central NCA as a salt-rich basin from a structural perspective. This exercise is particularly important because despite decades of research, to date there has been no attempt to systematically investigate the structural architecture of the area. In this contribution we present three regional cross-sections across the central NCA with which we aim to illustrate, for the first time, the structural implications of two competing structural scenarios:

1. The conventionally accepted scenario of significantly allochthonous Hallstatt units, with salt tectonics limited to the most distal NCA margin.
2. An alternative scenario with the presence of intra-platform salt tectonic structures. In this latter scenario we further explore the implications of deposition of the Hallstatt units above salt structures in intra-platform seaways, equivalent to the model of Oravecz et al. (2023) and recovering the concept of Hallstatt seaways of Zankl (1967).

## Regional cross-sections

### Background considerations

Three cross-sections are presented here (Figs. 3, 4a): Section 1 displaying the relationship of the Lammertal Zone to the Osterhorngruppe and Tennengebirge platform blocks; Section 2 displaying the relationship of the Altaussee Zone to the Singereben and Dachstein platform blocks; and Section 3 displaying the relationship of the Bad Mitterndorf Zone to the Totengebirge and Grimming platform blocks. To adequately represent these relationships, the cross-sections have been built in a NNE–SSW to N–S direction, orthogonal to the contacts between the platform blocks and their neighboring Hallstatt zones. Sections parallel to either the NW-directed or NE-directed transport directions documented since the Cretaceous (Linzer et al. 1995; Peresson and Decker 1997) would not cut across the contacts between the Hallstatt units and the neighboring Triassic platforms, rendering them of little use to understand the relationship between these units.

A further complication is implied by Miocene strike-slip. However, fault offset on individual fault systems is under 10 km within the NCA (Linzer et al. 2002; Levi et al. 2022) (Table 1). Since the E–W dimension of the main platform carbonate blocks and “Hallstatt zones” in the area is larger than this offset (Fig. 4a), strike-slip has not changed the relative positions observed in an N–S direction (Linzer et al. 2002; Frisch and Gawlick 2003). Therefore, even if out-of-plane motion precludes the possibility of balancing N–S cross-sections, their restoration is still useful to understand the evolution of the relative position of structural elements (platform blocks and “Hallstatt zones”) in an N–S direction through time.

In a highly unique approach, rather than presenting a single interpretation, we have chosen here to construct the three cross-sections to display two alternative scenarios: one scenario is compatible with the scenario of allochthony of the Hallstatt units; the second scenario seeks to represent a concept of relative autochthony of the Hallstatt units.

**Fig. 7:**
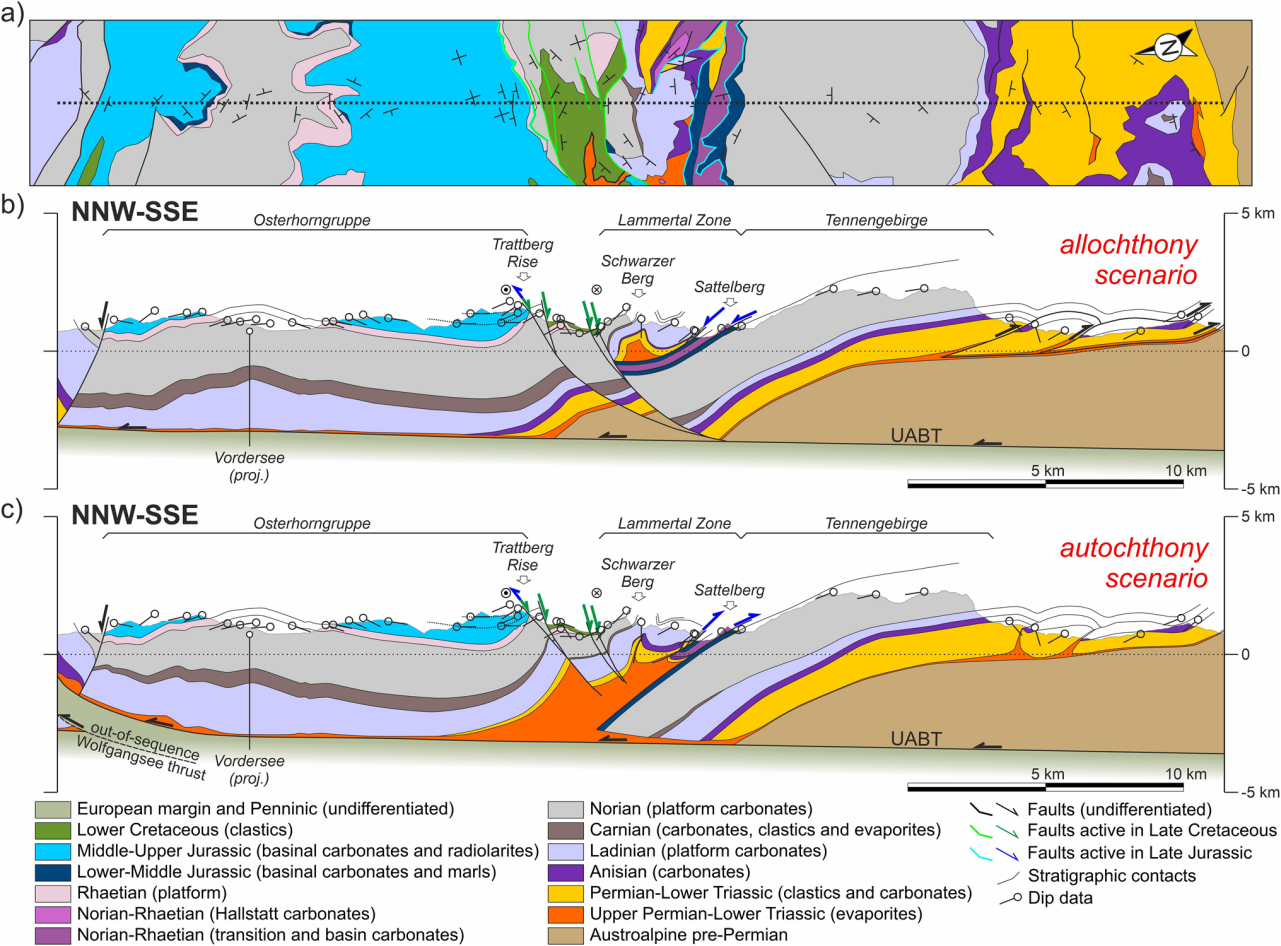
**a** Simplified geological map along Section 1. Adapted from Plöchinger (1987), Egger and van Husen (2003), and Krenmayr (2013). **b** Interpretation of Section 1 according to a scenario of allochthony of the Lammertal Zone units. **c** Interpretation of Section 1 according to a scenario of relative autochthony of the Lammertal Zone units. UABT: Upper Austroalpine basal thrust

**Fig. 8:**
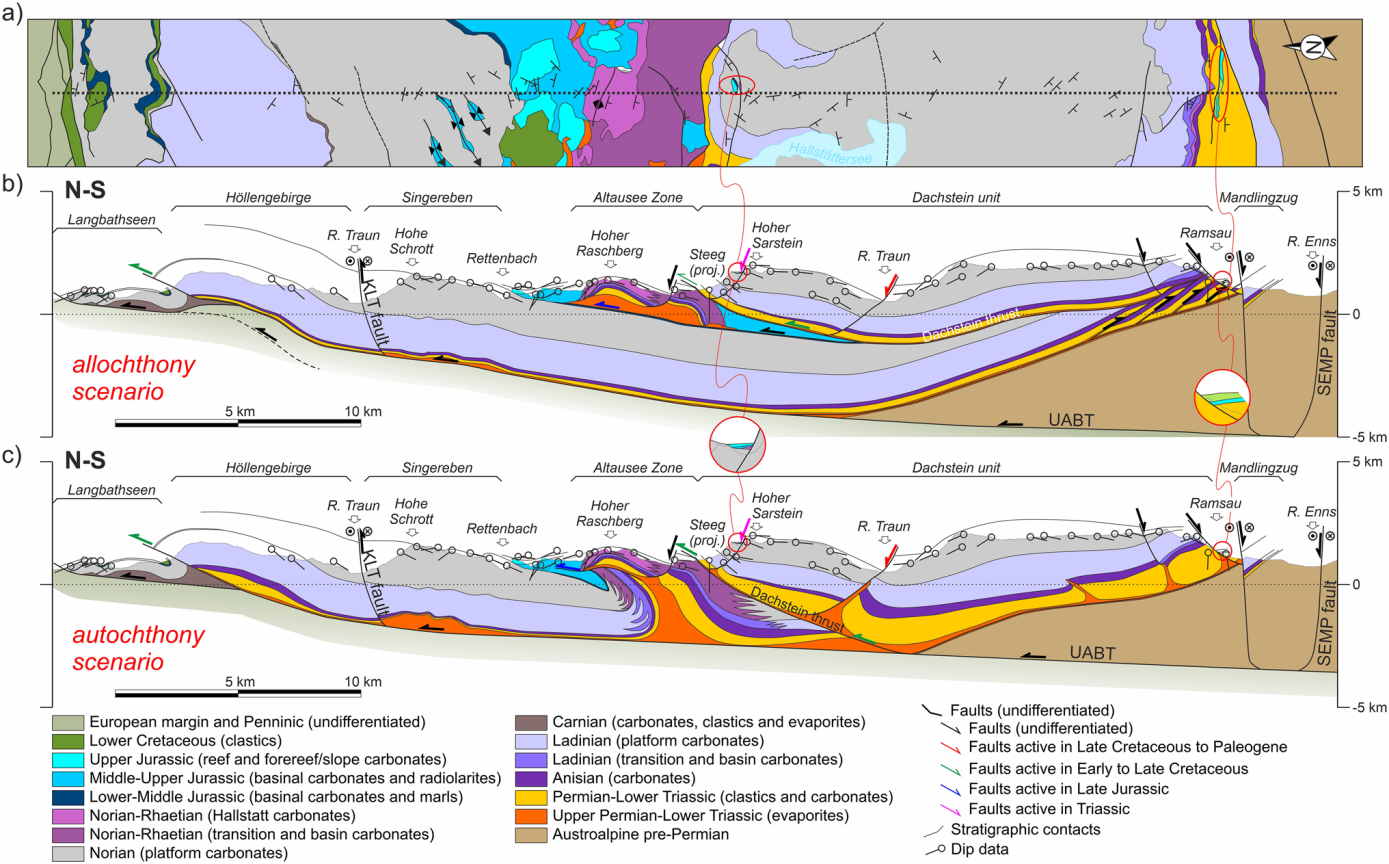
**a** Simplified geological map along Section 2. Adapted from Schäffer (1982), Mandl and Matura (1995), and Egger (1996). **b** Interpretation of Section 2 according to a scenario of allochthony of the Altaussee zone units. **c** Interpretation of Section 2 according to a scenario of relative autochthony of the Altaussee zone units. The insets show details of the Hoher Sarstein and Ramsau areas (see text for details). UABT: Upper Austroalpine basal thrust

**Fig. 9:**
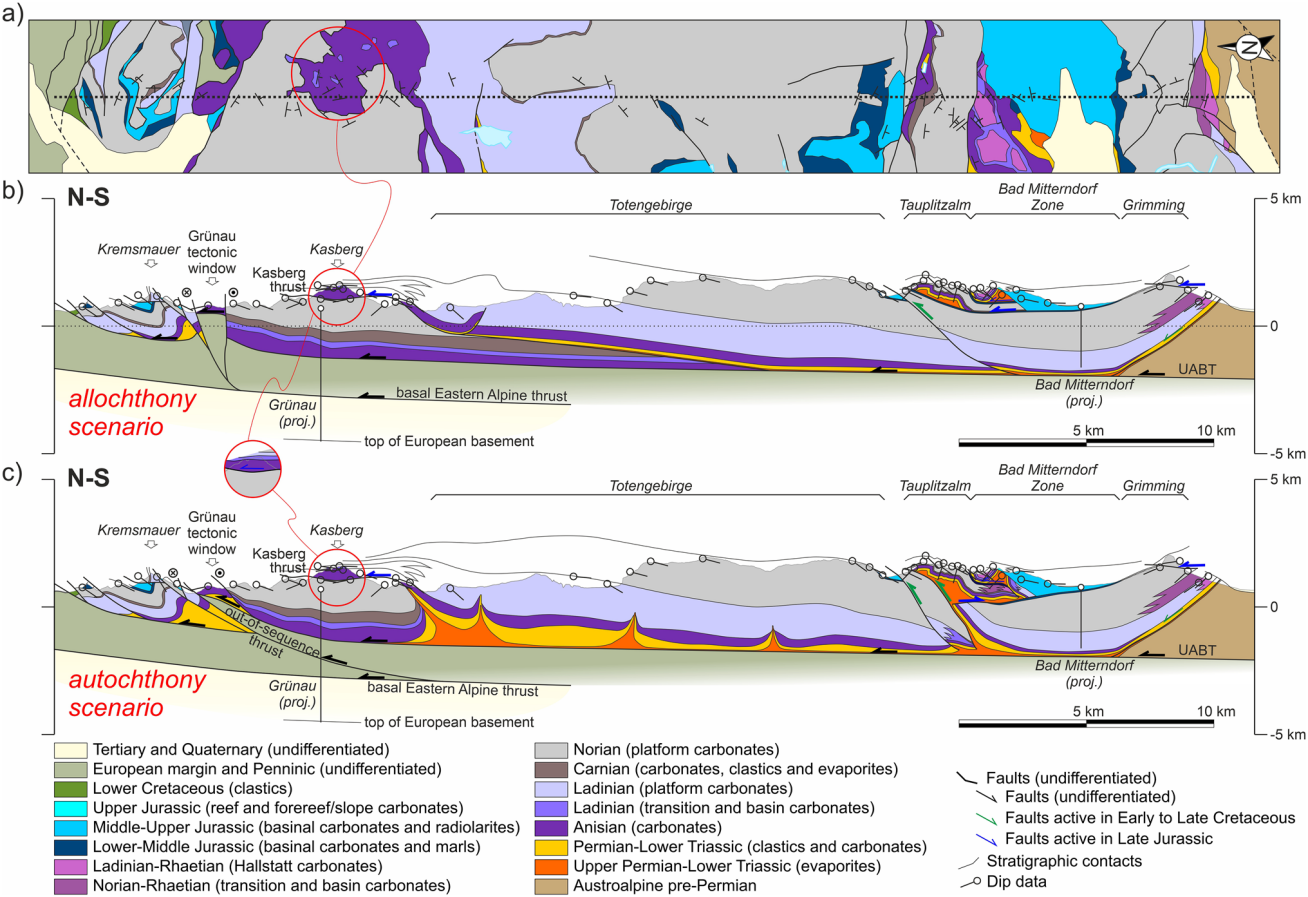
**a** Simplified geological map along Section 3. Adapted from Egger and Van Husen (2007), Moser (2014), and Kreuss (2020). **b** Interpretation of Section 3 according to a scenario of allochthony of the Bad Mitterndorf zone units and absent salt in the Kasberg thrust. **c** Interpretation of Section 3 according to a scenario of relative autochthony of the Bad Mitterndorf zone units and a salt-fed thrust model for the Kasberg thrust. The inset shows a detail of the Kasberg hanging wall (see text for details). UABT: Upper Austroalpine basal thrust

In the allochthony scenario, Triassic units (both shallow-water and deep-water carbonates) resting on Jurassic rocks are interpreted to have been transported from south of the NCA southern margin (Figs. 7b, 8b, 9b; cf. Figure 4b). In this scenario, it is interpreted that the Triassic platform units formed a single uninterrupted platform across the entire NCA, with stratigraphic thickness of the Triassic being constant or varying only gradually. In contrast, the allochthonous Triassic units, which are interpreted to have been deposited tens of kilometers south of their present location (Fig. 4d), have highly variable thickness. No significant volumes of Permo-Triassic evaporites are interpreted to have existed beneath the Triassic platform units.

The autochthony scenario assumes that Permo-Triassic salt was abundant across the central NCA and that Triassic platform units were deposited in minibasins (as proposed by Strauss et al. 2021). Triassic strata in these minibasins are interpreted to present variable thickness over relatively short distances due to halokinetic influence (e.g., stratigraphic thickness changes in Fig. 6). Synchronously, the Hallstatt units were deposited in deeper waters, atop salt structures located between the shallow-water platform minibasins, and also with variable thickness. Shortening of the salt bodies underlying the Hallstatt units led to basinal units thrusting onto their neighboring platforms. This scenario requires less total shortening than the allochthony model but in contrast interprets some structures to record south-directed thrusting (i.e., backthrusts) (Figs. 7c, 8c, 9c).

Both scenarios concur that the NCA (and their trailing pre-Permian basement) are at present thrust over thrust sheets derived from the Penninic Ocean and the European continental passive margin, as shown by numerous wells drilled in the NCA (Geuterbrück et al. 1984; Brix and Schultz 1993). The Upper Austroalpine basal thrust (UABT) at the base of the NCA units is interpreted to be of low angle and drawn at ~ 1–2° dip toward the hinterland (Figs. 7, 8, 9), similar to that of Linzer et al. (1995). This is consistent with the relatively steady topography across the central NCA, from foreland to hinterland (Fernandez et al. 2022), and is consistent with relatively tabular structure at depth observed on seismic profiles Alp01 and EASI (Brückl et al. 2007; Hetényi et al. 2018).

The Miocene strike-slip fault systems (namely the KLT and SEMP faults, Fig. 5a) are interpreted to be detached along the Alpine thrusts (Linzer et al. 1995, 2002). In contrast, both the Wolfgangsee and Windischgarsten fault systems uplift Penninic and distal European margin units (Plöchinger 1964; Linzer et al. 1995, 2002; Peresson and Decker 1997). These fault systems are therefore interpreted to be rooted below the UABT (Figs. 7, 9).

### Section 1: Osterhorngruppe–Lammertal–Tennengebirge

The westernmost section crosses the Lammertal Zone (Fig. 4a), a NW–SE trending belt of outcrops of Permo-Triassic evaporites and clastics, Anisian carbonates, Hallstatt units, and a large block (Schwarzer Berg) of Triassic platform (Fig. 7). The Lammertal Zone is bounded by Triassic platform carbonate blocks of the Osterhorngruppe to the north and the Tennengebirge to the south. The contact between the Lammertal Zone units and the Tennengebirge is interpreted to be either a north-directed structure for the allochthony scenario (Fig. 7b) or a south-directed backthrust for the relative autochthony scenario (Fig. 7c). The relative autochthony scenario assumes that the Schwarzer Berg is the southern prolongation of the Osterhorngruppe Triassic platform, at present cut off and offset from it by Cretaceous age faults (Fig. 7c). This scenario interprets that the remnants of a Triassic salt structure are partly preserved below the Lammertal Zone. In contrast, in the allochthony scenario the Schwarzer Berg is the uppermost allochthonous unit (Gawlick and Missoni 2019; Ortner et al. 2022), resting structurally on a sliver of Hallstatt units and Jurassic rocks (the Sattelberg unit) (Fig. 7b). The Tennengebirge and Osterhorngruppe are interpreted in this scenario to have been a continuous block of platform that is offset at present by Cretaceous age faults (Fig. 7b).

Related to the difference in interpreted relevance of salt in the structural configuration, the structure of the Lower Triassic south of the Tennengebirge (the Werfener Schuppenzone; Tollmann 1976b; Neubauer and Genser 2018) is interpreted to consist of either imbricate thrusts in the allochthony scenario (Fig. 7b) or of squeezed diapirs with upturned flanks in the relative autochthony scenario (Fig. 7c).

Both cross-sections interpret the presence of a wedge of pre-Permian Austroalpine basement rocks under the Triassic of the Tennengebirge (Fig. 7b, c). This wedge is the northern prolongation of the pre-Permian that crops out along the southern edge of the NCA (Figs. 3, 7a). The northern end of both sections coincides roughly with the location of the Wolfgangsee fault system. Although this system is in no way affected by the different scenarios of Hallstatt unit emplacement, the interpretation of this system has been made to honor a high-angle faulting scenario (Linzer et al. 1995, 2002; Peresson and Decker 1997) (due to their sub-vertical nature, the faults do not show up on Fig. 7b) or an alternate out-of-sequence (low-angle) thrust scenario (Fig. 7c). The out-of-sequence scenario requires thinning of the Triassic in the hanging wall which is easier to account for in the relative autochthony scenario. The interpretation of the northern segment of the cross-section is supported by the Vordersee well (Geutebrück et al. 1984; Egger et al. 2009). For simplicity, a thin sliver of Jurassic and Cretaceous encountered by the Vordersee borehole below the Triassic succession (interpreted to be part of the frontal most NCA thrust sheet in this area) has not been differentiated from the UABT footwall units. This implies that the UABT is in fact somewhat deeper than shown in Fig. 7, but this has no impact whatsoever on the interpreted shallower structure and would add unnecessary complexity to the section.

### Section 2: Höllengebirge–Altaussee–Dachstein

The central section crosses the Altaussee Zone (Fig. 4a), an area in which Triassic Hallstatt units crop out extensively (Fig. 8). On this section the Altaussee units rest on the Singereben–Höllengebirge platform block and are thrust over by the platform block of the Dachstein thrust sheet. In the allochthony scenario, the Altaussee units are considered to be rootless and highly allochthonous, originating south of the Dachstein block (Mandl et al. 2012) (Fig. 8b). This interpretation makes it necessary to extend the Singereben block under the Altaussee Zone south into the footwall of the Dachstein thrust, implying both platform blocks were initially a through-going unit.

In the relative autochthony scenario, in contrast, the Altaussee units are interpreted to be the roof that originally sat between the Singereben and Dachstein platform blocks and has been thrusted onto the Singereben block (Fig. 8c). Although displacement at the base of the Altaussee Zone cannot be determined, the interpretation in this scenario opts for a relatively conservative shortening amount (under 10 km). By doing so, the Singereben platform block is interpreted not to extend under the Altaussee Zone significantly. The result is a shallower UABT (Fig. 8c) than that in the allochthony scenario (Fig. 8b).

As in Section 1, the southernmost Triassic platform block (Dachstein unit) is interpreted to be underlain by a wedge of pre-Permian Austroalpine basement (Fig. 7b, c). The nature of faulting along the southern end of Section 2 (SEMP fault and its splays) is not the objective of this contribution and represented schematically in both scenarios as transtensional due to the offset relationships across the faults (Fig. 7b, c). The northern end of the section is interpreted very similarly in both scenarios too. Only a minor difference has been introduced in the form of a sub-UABT thrust below the Höllengebirge in the allochthony scenario (Fig. 8b) to preserve the constant-thickness condition of the allochthony scenario.

This section shows two noteworthy features, highlighted with insets, that are discussed in the restorations. One is the presence of Hallstatt Limestone directly overlying the Triassic platform succession on the Hoher Sarstein (leading edge of the Dachstein thrust sheet). The second feature are exposures of Upper Jurassic reefal limestone and Upper Cretaceous clastics along the trailing edge of the Dachstein thrust sheet, in the area of Ramsau (Mandl 1998; Mandl et al 2014). These rocks lie directly on Lower Triassic clastics, and lie between the Dachstein unit to the north and the Mandlingzug to the south. The Mandlingzug itself is a structural block along the SEMP fault system with a stratigraphy that differs from the neighboring Dachstein unit and has been interpreted to derive from west of the study area (e.g., Frisch and Gawlick 2003).

### Section 3: Kasberg–Totengebirge–Bad Mitterndorf

The eastern section (Fig. 9) crosses the Bad Mitterndorf Zone (Fig. 4a), an area in which Triassic Hallstatt units crop out in a patchy arrangement in association with Upper Jurassic strata, and the Kasberg Middle Triassic basin (Fig. 4a), an area with Middle Triassic basinal units. The Bad Mitterndorf Zone lies between the Grimming and Totengebirge platform blocks, whereas the Kasberg basin is interpreted to have developed as an intra-platform basin within the Totengebirge platform block. The Kasberg basin (and its condensed stratigraphy) is preserved in the hanging wall of the Kasberg (Totengebirge) thrust (inset in Fig. 9), directly overlying a thick Upper Triassic platform in the footwall.

The interpretation on this cross-section is supported by two deep boreholes: the Grünau-1 and the Bad Mitterndorf TH-1 wells (Hamilton 1989; Schmid et al. 2003). The Grünau-1 drilled the UABT thrust, the underlying allochthonous European margin and Penninic units, and the autochthonous foreland above the European basement. The Bad Mitterndorf TH-1 well in turn drilled a thick Middle to Upper Triassic succession that forms part of the Grimming unit (Fig. 7b, c) but failed to reach the basal UABT thrust.

In the allochthony scenario, the Tauplitzalm units are interpreted to be an allochthonous unit (along with the Bad Mitterndorf Zone) based on the highly reduced thickness of the Triassic (Tollmann 1960; Schöllnberger 1973; Moser 2014). Along with the Bad Mitterndorf Zone units, they are interpreted to have been emplaced in the Jurassic onto the underlying Totengebirge–Grimming unit that is relatively constant in thickness (Fig. 9b). In order to preserve stratigraphic thickness of the Totengebirge block toward the north, a very low-angle thrust ramp has been introduced that feeds into the Kasberg thrust (part of the Totengebirge thrust of Tollmann 1976b). The low angle of the thrust ramp could be explained by the high obliquity of the section to the SE-dipping, NW-directed thrust.

In the autochthony scenario, the Tauplitzalm constitutes the southern prolongation of the Totengebirge (similar to the relationship between the Schwarzer Berg and the Osterhorngruppe in Section 1). The Tauplitzalm unit is thrust northwards onto the Totengebirge block, and the rocks of the Bad Mitterndorf Zone are backthrust onto the Grimming unit (Fig. 9c). In this scenario, the Totengebirge platform is represented with variable thickness and the Kasberg thrust is interpreted to be a salt-fed thrust (Fig. 9c).

Finally, two possible alternative interpretations are presented for the Grünau tectonic window (part of the Windischgarsten fault system) (Fig. 9b, c). One interpretation contemplates a strike-slip system rooted in the basal Alpine thrust (Fig. 9b). The alternative interpretation assumes the presence of an out-of-sequence thrust cutting up-section from the basal Alpine thrust (Fig. 9c), akin to the structural sketch of Hamilton (1989).

## Cross-section restoration

The three presented cross-sections (Figs. 7, 8, 9) fit the available outcrop and borehole data with two strongly contrasting structural interpretations. Due to significant out-of-plane motion, these cross-sections cannot be balanced. However, as discussed above, they can be restored to observe the evolution of the relationships between adjacent blocks through time. This restoration elucidates important structural implications of the two alternative scenarios that are difficult to surmise from the present-day cross-sections.

### Restoration methodology

The objective of sequential restoration is to understand the geometric and tectonic relationships of the main structural elements of the central NCA through time. The restorations have intentionally left out elements north of the Wolfgangsee and Windischgarsten systems and south of the SEMP system to reduce complexity related to these fault systems. The restorations performed assume that tectonic shortening is north directed. Although this is not necessarily correct, the aim is merely to focus on the evolving relationships (relative position) between structural elements.

**Fig. 10:**
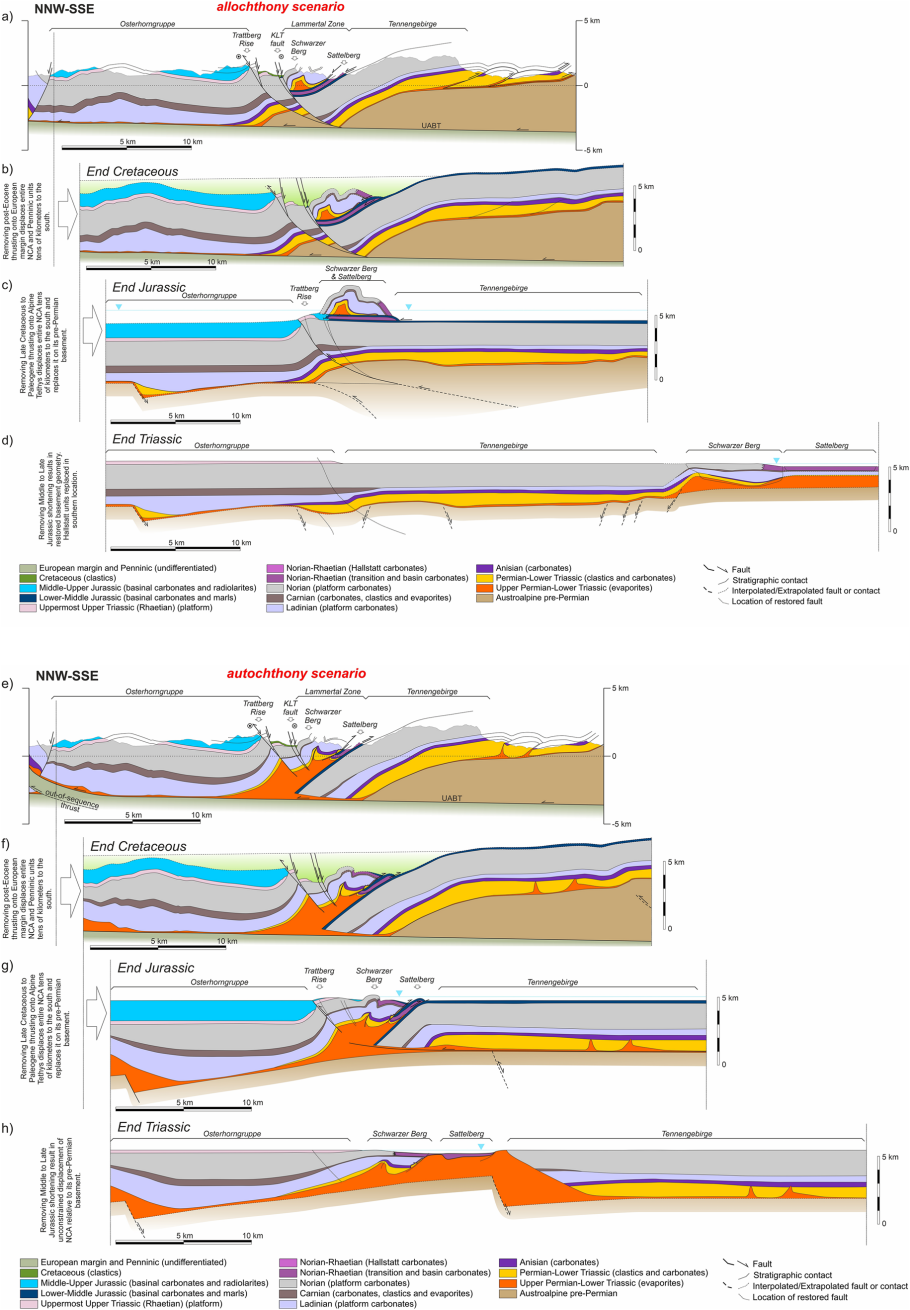
Sequential restoration of Section 1 for the allochthony (**a**–**d**) and autochthony (**e**–**h**) scenarios. See text for details

**Fig. 11:**
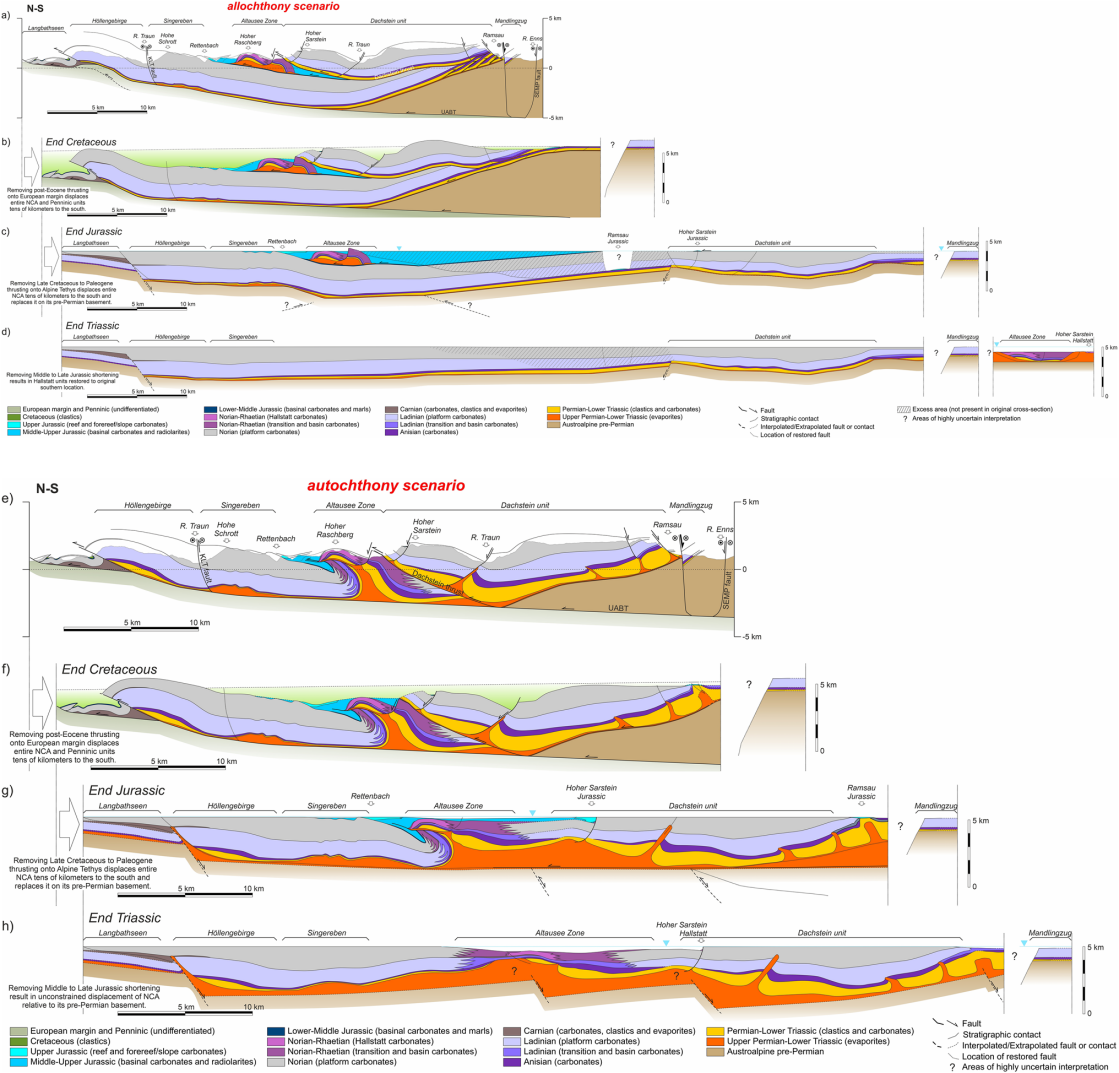
Sequential restoration of Section 2 for the allochthony (**a**–**d**) and autochthony (**e**–**h**) scenarios. See text for details

**Fig. 12:**
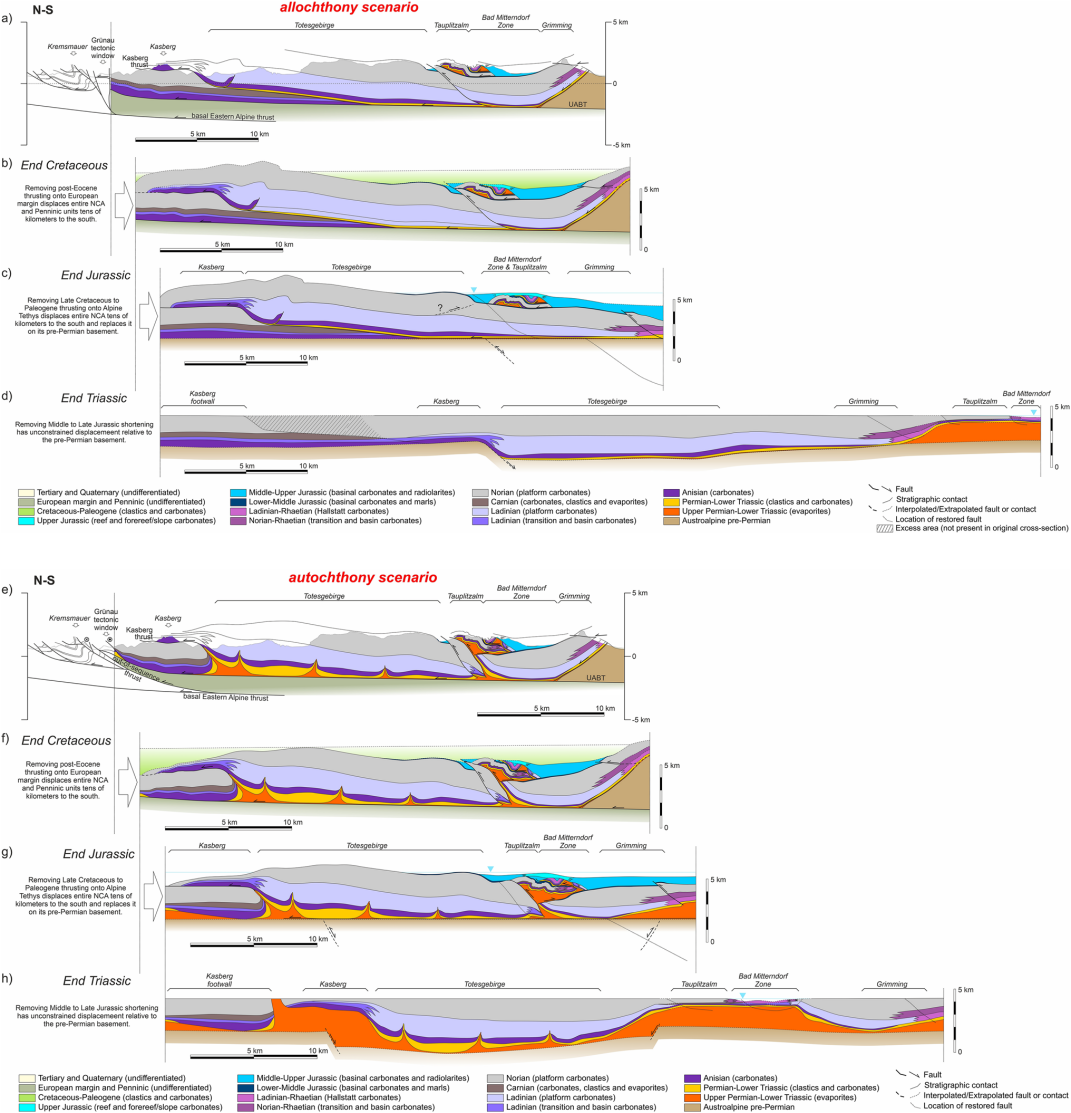
Sequential restoration of Section 1 for the allochthony (**a**–**d**) and autochthony (**e**–**h**) scenarios. See text for details

The time of activity of structures (faults, folds) has been derived from our own work and the work of previous authors (Tollmann 1981; Mandl 2000; Linzer et al. 1995, 2002; Krische and Gawlick 2015; Ortner 2017; Gawlick and Missoni 2015, 2019; Levi 2023), and is summarized in Table 1. Three restored time steps have been generated for each cross-section (Figs. 10, 11, 12):

- End of the Late Triassic (Figs. 10d, h, 11d, h, 12d, h): this marks the end of the development of Triassic platforms in a passive margin setting. At this time, the NCA units are resting on their corresponding Austroalpine basement. Faults in the basement have been interpreted in order to accommodate thickness differences in the Triassic. The top of the Triassic platforms is interpreted to have been near sea level, whereas the Hallstatt units were deposited in depths of around 500 m. In the allochthony scenario, the reconstructed paleogeography places all the Hallstatt units south of a single Triassic carbonate platform (as in Fig. 4b), whereas in the relative autochthony scenario, the Hallstatt basins are interpreted as intra-platform lows (as in Fig. 4c);
- End of the Jurassic (Figs. 10c, g, 11c, g, 12c, g): this moment shows the Hallstatt Zones already emplaced onto the Triassic platform blocks. This is the critical time step to understand the structural relationship between the Triassic shallow- and deep-water carbonates. At this time, the NCA units continue to sit atop their Austroalpine basement without any shearing (in the allochthony scenario) or with some decoupling along the Permo-Triassic evaporites (relative autochthony model). In both models it is interpreted that shortening was accompanied by inversion of extensional basement faults;
- End of the Cretaceous (Figs. 10b, f, 11b, f, 12b, f): this moment shows the completed emplacement of the Early Cretaceous structures in the NCA (namely, the Dachstein thrust sheet) and pre-dates the youngest phase of Alpine deformation. At this time, the NCA units had been decoupled from their underlying basement (except along their trailing edge) and thrust over the Penninic (Alpine Tethys) units. This event of northward thrusting is interpreted to transport the NCA thrust sheets mostly as a coherent block, with the local development of extensional basins (Wagreich and Decker 2001; e.g., Fig. 7b, f). A gently foreland-dipping (1°) top of the Gosau Gp, infilling pre-existing topography and extensional depocenters, has been interpreted. This situation pre-dates post-Gosau deformation associated to thrusting and strike-slip (e.g., Peresson and Decker 1997; Levi 2023).

The restoration has been performed for both scenarios: that of allochthony (Figs. 10a–d, 11a–d, 12a–d) and that of relative autochthony (Figs. 10e–h, 11e–h, 12e–h) of the Hallstatt units. The restorations have been performed by unfolding of fault blocks using a flexural flow algorithm (Griffiths et al. 2002), and the fault block restoration method discussed in Lingrey and Vidal-Royo (2015). The restoration has resulted in gaps and overlaps between fault blocks. Where these were under 5% of section length or related to eroded segments, they have been edited in the presented results. In contrast, significant gaps or overlaps (in excess of 15% of section length) are highlighted on the sections. In spite of the non-balance character, extreme changes in cross-sectional area may still indicate problems in the interpretation.

### Results of the restoration and implications

It is not our objective here to discuss the restorations in Figs. 10, 11, 12 in detail. We concentrate instead on the higher-order features or critical elements that can be considered independent of the restoration strategy, and that are relevant to the evolution of the central NCA and for the discussion on the origin of the Hallstatt units.

#### Basement structure

All sections, irrespective of the scenario, require a stepped geometry (i.e., faulted basement) at the base of the Triassic succession (Figs. 10d, h, 11d, h, 12d, h). No attempt has been made to restore the cross-sections beyond the end of the Triassic. However, it is remarkable that both the Middle and Upper Triassic platform units present significant thickness changes from north to south. The largest change in thickness occurs between the Langbathseen and Höllengebirge units (Section 2, Fig. 11d, h), historically considered the boundary between the Bajuvaric and Tirolic thrust systems (Tollmann 1976b). In the relative autochthony scenario this can be accounted for by either halokinesis (evacuation of salt under the Middle to Upper Triassic carbonate platform minibasins, as in Strauss et al. 2021), by ongoing faulting in the basement, or a combination of both. In the case of the allochthony scenario, basement faulting throughout the Late Triassic is the only possibility to generate the observed thickness changes (as documented in neighboring provinces by Bertotti et al. 1993 and Héja et al. 2018).

Another surprising basement-related feature appears in the allochthony scenario of Section 3: the development of the Totengebirge Middle Triassic platform in the hanging wall (subsided) block of an extensional fault and the Kasberg basin in its footwall (Fig. 12d). This incongruent arrangement, already exposed by previous authors and highlighted by Kenter and Schlager (2009), is less problematic in the relative autochthony scenario where the Totengebirge platform developed as a minibasin and the Kasberg basin above inflated salt (Fig. 12h).

Evolution of the basement from the Late Triassic to present is mostly unconstrained. However, both the allochthony and relative autochthony scenarios require some amount of basement-involved deformation (i.e., basement uplift) during the Jurassic (Fig. 10c, g) to account for the difference in accommodation space between the Osterhorngruppe (with nearly 1000 m of Middle to Upper Jurassic strata) and the Tennengebirge (apparently mostly barren). It is possible, though, that Jurassic strata above the Tennengebirge may have been eroded (see discussion on Cretaceous metamorphism below). Likewise, the emplacement of the Dachstein–Grimming and Höllengebirge thrust sheets in the Lower Cretaceous has been interpreted to involve basement in their trailing edge as the simplest solution kinematically (an alternative later emplacement of the basement as a wedge is possible but not necessarily supported by any evidence).

#### Late Triassic configuration

As is implied in their definition, the allochthony scenario and the relative autochthony scenario restorations differ most significantly in the Triassic. Whereas in the allochthony scenario Upper Triassic basins are found exclusively to the south of a broad (many tens of kilometers) platform (Figs. 10d, 11d, 12d), in the autochthony scenario Hallstatt units deposited in seaways (bathymetric lows) located above salt structures between salt-floored minibasins (Figs. 10h, 11h, 12h). This mirrors the configuration of the Middle Triassic basins (Kasberg in Fig. 12h). The cross-sectional area of the Permo-Triassic evaporites in the Triassic restored state implies that their thickness was in the order of at least 700 to 1000 m prior to evacuation through minibasin formation, a thickness that is in line with that proposed by Leitner and Spötl (2017) or cited by Schöllnberger (2021) and still less than the 3 km proposed by Strauss et al. (2023) in the eastern NCA.

#### Late Jurassic tectonism and sedimentation

The Middle to Late Jurassic deformation phase brought about the emplacement of the deep-water Hallstatt units onto the Triassic platform blocks. This process is solved differently in the relative autochthony and allochthony scenarios, based on the diverging paleogeographic interpretations. Irrespective of the scenario, however, both imply some form of tectonic activity and are thus consistent with the widespread evidence for instability deposits during this time (Gawlick et al. 1999, 2007).

In the relative autochthony scenario, structural juxtaposition of Triassic basinal and platform rocks is achieved by widespread shortening across the central NCA, with thrusting and backthrusting of Triassic basinal units onto their neighboring carbonate platforms (Figs. 10g, 11g, 12g). Deformation concentrated above pre-existing salt structures and led to the development of positive relief above these structures.

The juxtaposition of basinal and platform units is explained in the allochthony scenario through longer-distance allochthony. However, low-angle emplacement of the Hallstatt units in the allochthony also requires thrusting involving the basement of the NCA to account for Jurassic basin geometries: the Trattberg thrust (Fig. 10c) and a backthrust under the Rettenbach (Fig. 11c).

**Fig. 13:**
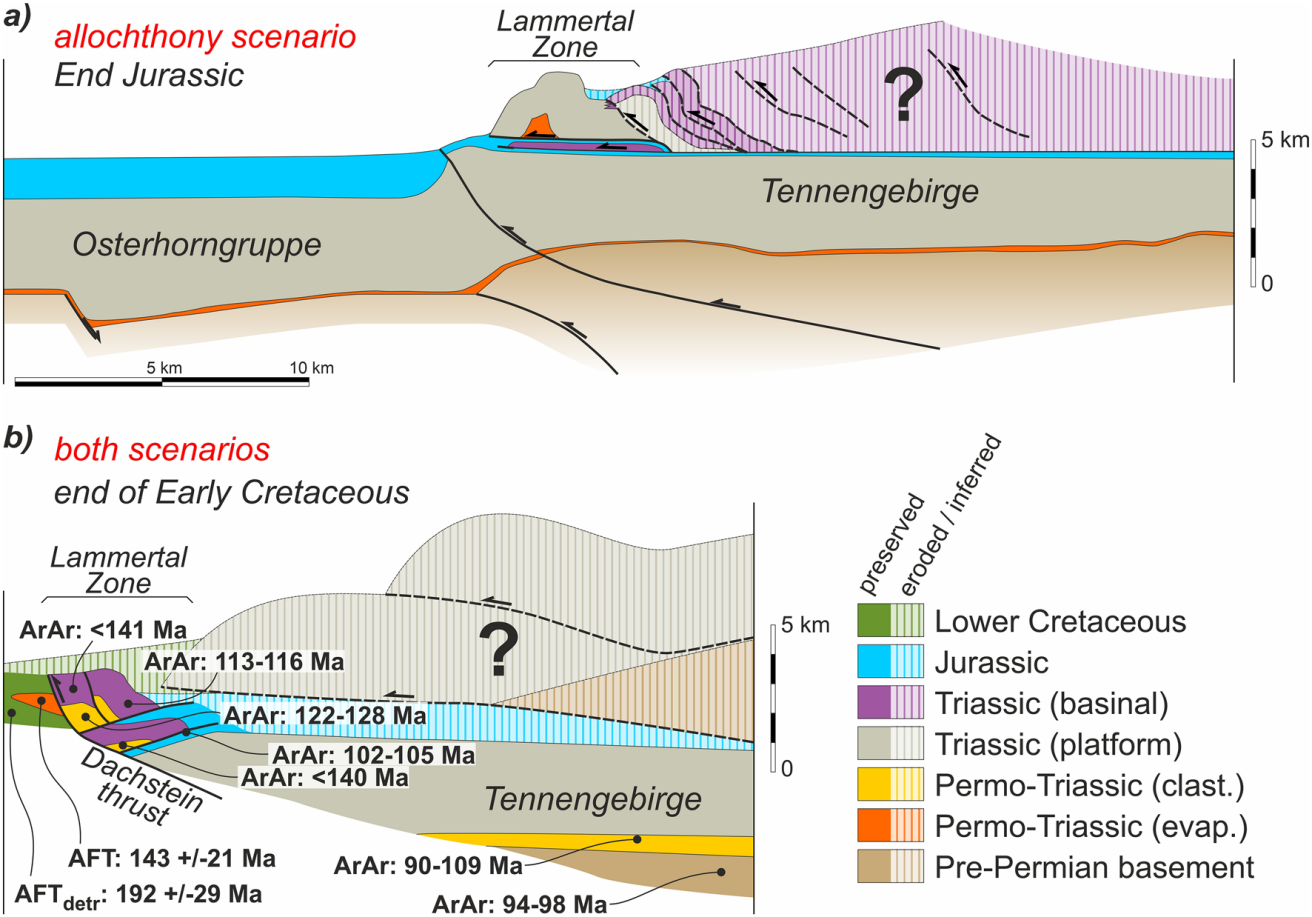
**a** Schematic representation of the potential geometry of a wedge of thrust imbricates to account for the emplacement of the Lammertal Zone units (cf. Figure 10c). Late Jurassic synthrusting strata would also be expected to be involved in the thrust imbricates. **b** Schematic interpretation of the possible structure of the Lammertal–Tennengebirge area (southern end of Section 1) at Middle Cretaceous. Ar^39^/Ar^40^ ages (Frank and Schlager 2006) are for illite and sericite grains newly formed or reset due to low grade metamorphic event. AFT (apatite fission track) ages (Hejl and Grundmann 1989) are from detrital (AFT_detr_) apatite in Lower Cretaceous sediments and from dolerite contained in the Moosegg Permo-Triassic allochthonous salt (Fig. 5b.1, Table 1) extruded in the Lower Cretaceous, and linked to thrusting of the Dachstein–Schwarzer Berg (Schorn and Neubauer 2011)

The mechanisms necessary for long-distance allochthony of the Hallstatt units during the Late Jurassic and their implications are not discussed here. On the cross-sections emplacement of these units is represented as isolated gravitationally slid masses. Alternatives to this model (fold-and-thrust system or accretionary wedge; Fig. 13a) do not substantially change the relationships represented in the restored cross-sections.

#### Cretaceous to Neogene shortening

After the phase of Late Jurassic shortening, the evolution of both the relative autochthony and allochthony scenarios is interpreted to be the same (Fig. 10b, f, 11b, f, 12b, f). Cretaceous shortening is depicted in the restorations as one single event despite constituting a series of events. Firstly, the Dachstein thrust sheet was emplaced during the Early Cretaceous. At that time, the central NCA units were still overlying their original Austroalpine basement, and it was likely partially getting involved in deformation. A posterior (still Early Cretaceous) phase of deformation involves emplacement of the Höllengebirge thrust sheet onto the Langbathseen units (Fig. 11b, f) and initial northward thrusting of the Langbathseen units. Then, during Late Cretaceous thrusting proceeded to transport the entire NCA northwards onto the Penninic Ocean units, decoupling most of the NCA from their original basement. Although the northward emplacement of the NCA units onto the Penninic implies major amounts of displacement (many tens of kilometers at least), relatively limited deformation occurred within the NCA (folding and small-scale thrusting; Levi 2023). Also partly coeval with these events is the development of the Upper Cretaceous fault-bounded depocenters (Wagreich and Decker 2001; Fernandez et al. 2022), extensional faulting between the Osterhorngruppe and the Schwarzer Berg (Fig. 10b, f), and extension within the thrusted Dachstein unit (Fig. 11b, f) (Fernandez et al. 2022). Although Cretaceous faulting in the NCA is associated with transtension (Wagreich and Decker 2001; Froitzheim et al. 2012), a possible link to salt-cored deformation (cf., Fernandez et al. 2022) has been incorporated in the autochthony scenario (Figs. 10f, 11f).

Imbrication of the Penninic units and thrusting of the entire stack over tens of kilometers northwards onto the European margin during the Cenozoic (Figs. 10a, e, 11a, e, 12a, e) once again generated relatively limited amount of deformation within the central NCA.

Neogene strike-slip deformation is not represented on the restored sections as the amounts of displacement did not significantly alter the key target of this study; the relative position between the Triassic basin and platform units remained mostly fixed since Late Jurassic times.

#### Allochthony of the Dachstein thrust sheet

The restored position of the Dachstein thrust sheet (Fig. 11c, d, g, h) is presented differently depending on the interpretation of the present-day structure. In the model supported by Schweigl and Neubauer (1997) and Mandl (2000) (and previous authors therein), the basement at present underlying the Dachstein is interpreted to correspond to the Höllengebirge–Singereben thrust sheet. This requires an original position of the Dachstein thrust sheet further to the south of this basement in its restored position (Fig. 11d), making this unit highly allochthonous. This restored geometry requires interpreting the presence of a now-eroded segment of the Höllengebirge thrust sheet (striped area in Fig. 11c, d). A scenario in which the Dachstein thrust sheet is less allochthonous (e.g., as proposed by Frisch and Gawlick 2003), represented in the relative autochthony scenario, does not require the introduction of this eroded rock volume.

#### Other noteworthy elements

Other than the above observations, it is important to highlight the following:

1. The relative autochthony scenario implies the presence of significant amount of Permo-Triassic evaporites in the subsurface of the central NCA, consistent with growth geometries in the Dachstein Limestone (Fig. 6) and evidence of the relatively autochthonous position of the Hallstatt and Wurzeralm diapirs (Fernandez et al. 2021; Kurz et al. 2023).
2. The relative autochthony scenario restoration (Figs. 10e–h, 11e–h, 12e–h) can account for the observed present-day geometries while, surprisingly, preserving cross-sectional area during restoration. This could imply that (other than Neogene strike-slip) out-of-plane motion might have been constant along the length of the sections (therefore not affecting balance). In contrast, the allochthony scenario requires the removal of significant volumes of cover units (striped areas in Figs. 11c, d, 12d). Whether these area changes can be accounted for by out-of-plane motion has not been explored.
3. The relief of the Sattelberg and Schwarzer Berg at Late Jurassic times in the allochthony scenario is unnaturally prominent (Fig. 10c). A geometry of these units as part of an imbricate tectonic wedge (Fig. 13a) is more geologically plausible. Notwithstanding, Gawlick et al. (2009) found no evidence of reworking of materials from these allochthonous units in the Upper Jurassic of the Osterhorngruppe.
4. The shallow-water Upper Jurassic strata of the Hoher Sarstein and Ramsau (Fig. 11c, g) have two very contrasting origins in both scenarios. The Hoher Sarstein reef slope Jurassic (Schlagintweit and Gawlick 2006) sits atop Hallstatt and Dachstein Limestones (Mandl 2003; Fernandez et al. 2022). In the allochthony scenario, the Hallstatt Limestone block is considered to have been emplaced onto the Dachstein Limestone of the Dachstein thrust sheet in the Late Jurassic (Fig. 11c), whereas in the relative autochthony scenario it is interpreted to have been deposited above a drowned block of Dachstein Limestone (Fig. 11h; Fernandez et al. 2022). Upper Jurassic reef detritus in the first case would be interpreted to potentially come from an Upper Jurassic reef further west (e.g., Mt. Plassen reef; Mandl et al. 2012), in the second case they would be sourced from the reef developing atop the Altaussee Zone to the north. The Upper Jurassic of the Ramsau is more critical. In the relative autochthony scenario it is interpreted to have deposited above a squeezed diapir that grew during the Triassic with no significant roof (as in other instances in the central NCA; Fernandez et al. 2021; Kurz et al. 2023) (Fig. 11h). This accounts for the absence of Triassic platform below the Upper Jurassic. In contrast, the position of Upper Jurassic directly above Permo-Triassic strata cannot be accounted for in the allochthony scenario through deposition and would require either
  - extreme extensional faulting on a low-angle fault prior to the Late Jurassic (to eliminate the Middle to Upper Triassic succession) followed by uplift of the Permo-Triassic substrate by an unknown mechanism in the Late Jurassic or
  - deposition of the Upper Jurassic at a different location and posterior extreme extension on an unidentified low-angle fault to obtain the present-day juxtaposition.

## Discussion

### Allochthony versus relative autochthony: the role of salt tectonics in the NCA

The debate on the allochthony or relative autochthony of the Hallstatt units in the central NCA was mostly brought to a close by the contribution of Tollmann (1981) and the further elaboration by other authors, to its consolidation in its current form (Mandl 2000). Likewise, an alternative mode of emplacement of allochthonous Hallstatt units, as part of a now dismembered accretionary prism or thrust system, has been proposed (Gawlick et al. 1999; Missoni and Gawlick 2011; Gawlick and Missoni 2019; Strauss et al. 2023). However, to date, no structural representation of the full spatial and temporal significance of these models has been attempted. Neither has the relevance of salt tectonics been explored to date in the central NCA. Therefore, and based on the results presented above, we argue that the interpretation of the central NCA as a salt-rich province is compatible with a relatively autochthonous origin of the Hallstatt facies and with the key structural features of the area.

**Fig. 14:**
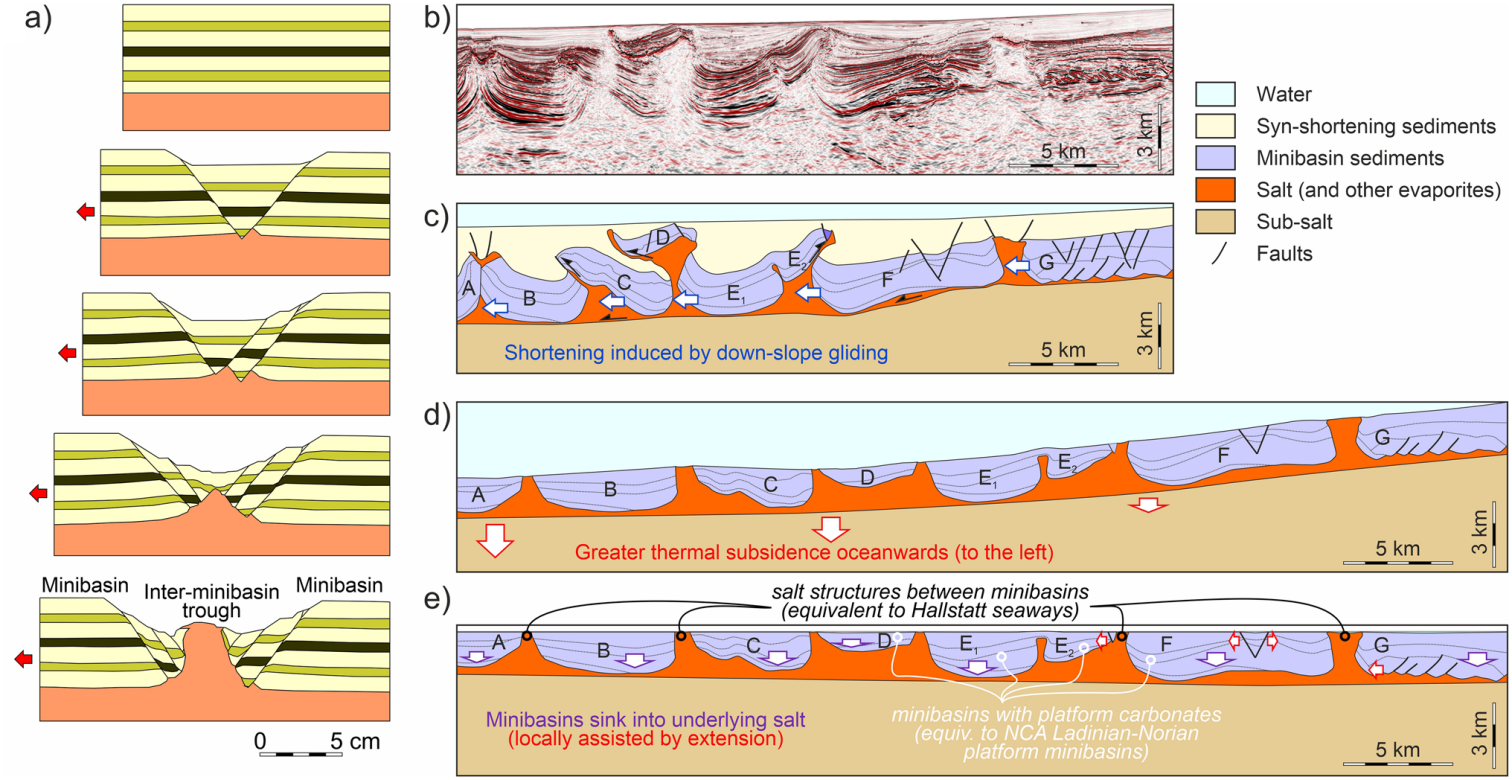
**a** Analogue sandbox model illustrating the negative relief above a diapir growing in extension. This model of growth could account for the presence of subsiding basins above salt structures within the Triassic shallow-water carbonate platform. After Vendeville and Jackson (1992) **b–e** Depth-migrated reflection seismic profile, from the offshore Kwanza basin (Angola) (courtesy of Sonangol), its interpretation (**c**) and restoration (**d–e**). Minibasins above the salt developed initially by subsidence into the underlying salt (**e**) and were eventually driven by differential thermal subsidence (**d**) into downslope gliding, which in turn led to shortening that concentrated on the pre-existing diapirs (**c**)

In particular, based on observations specific to the central NCA (as described above, e.g., Fig. 6), it is reasonable to propose that the Middle to Upper Triassic platforms of the central NCA developed as minibasins subsiding into a thick Permo-Triassic evaporite unit (similar to the model of Strauss et al. 2021 for the eastern NCA). In settings where minibasins develop under extension, diapirs between the minibasins will develop with negative relief (Vendeville and Jackson 1992; Fig. 14a). In such an arrangement, the Hallstatt seaways of Zankl (1967), as well as Middle Triassic basinal domains (e.g., Kasberg, Fig. 12h), could have developed above extending intra-platform diapirs. Such a model would provide an explanation for the shallow basin paradox discussed by Kenter and Schlager (2009). That is, basinal deposits in the central NCA would have developed above diapirs with negative relief (deep water), whereas shallow-water carbonates accumulated in subsiding minibasins but aggraded rapidly enough to prevent drowning. The presence of Middle and Upper Triassic platform detritus in the slope sediments of the Hallstatt units (Pistotnik 1972; Gawlick 1998; Hornung et al. 2007; Mette et al. 2019) and the transition of Hallstatt slope units into Hallstatt Limestones s.s. (Pistotnik 1974) indicates the syndepositional vicinity of the platform and Hallstatt domains. In spite of large carbonate productivity in the Triassic platforms, in this scenario, the platforms must not have prograded over the Hallstatt seaways. Indeed, seaways hundreds of meters deep and persisting over tens of millions of years have been documented within aggrading carbonate banks in tectonically quiescent areas (e.g., Eberli and Ginsburg 1987; Aubert and Droxler 1996). In the NCA the Hallstatt seaways may have been spared burial by prograding platforms due to ongoing extension (Fig. 14a) and strong bottom currents that exported excess carbonate production (cf. Betzler et al. 2009, 2015; Lüdmann et al. 2013, 2016). Strong current activity is recorded in the Hallstatt Limestone as condensed sections of red nodular limestones with ferromanganese hardgrounds, winnowed bivalve shell beds many meters thick, or unbedded bioturbated lime mudstones, that potentially represent sediment drifts (Diener 1926; Pistotnik 1974; Mandl 1984; Gawlick 2000; Hornung 2007; Hornung et al. 2007; McRoberts et al. 2008).

It is worth mentioning, that in contrast to the controversy surrounding the origin of the Hallstatt units, the accumulation of deep-water carbonate successions (Seefeld Fm) within the Upper Triassic platform domain of the Hauptdolomit in the western NCA has always been accepted (e.g., Brandner and Poleschinski 1986; Donofrio et al. 2003). The Hauptdolomit intra-platform basins may have been similarly related to a combination of extension and reactive diapir growth.

Shortening (from Jurassic onwards) of a salt-rich system would have led to the preferential re-activation of salt structures as thrusts (Fig. 14b–e). In such systems, the presence of backthrusts (as the Sattelberg backthrust on Section 1, Fig. 7c) is not uncommon (thrust of minibasin E onto minibasin F in Fig. 14c) and has even been previously proposed in the eastern NCA (backthrust of the Gamstein unit in Santolaria et al. 2022). One of the consequences of the re-activation of diapirs in shortening would be that the transitions between Triassic platforms and basins would have been the preferential loci of deformation. As pointed out by several authors (e.g., Pistotnik 1974; Frisch and Gawlick 2003), and as contained in the cross-sections presented (Figs. 7, 8, 9), complete transitions from the Hallstatt units into the Upper Triassic platform successions are mostly strongly tectonized. A “smoking gun” observation in favor of these transitions into an intra-NCA seaway has not yet been made. The lack of documented complete platform-to-basin transitions notwithstanding the structural model presented here in favor of a relative autochthonous origin of the Hallstatt units represents a framework with which to identify areas for future investigation. Furthermore, the nature of the platform-to-basin transitions in a relative autochthony scenario might also need to be reconsidered. The Hallstatt seaways proposed by Zankl (1967) were characterized by the presence of reefs along their northern margins (Fig. 4c). Reefs are, however, absent along their southern fringes. This may simply reflect the typical difference between windward (reefs) and leeward (carbonate sand bodies, platform shoulders) platform margins (cf. Hine et al. 1981).

Notwithstanding, these observations may (as has been done until now) also be explained within a “single platform—single Hallstatt facies belt” framework in combination with allochthonous emplacement.

### Structural inheritance in the central NCA

Irrespective of the interpreted origin of the Hallstatt units, the evidence for widespread Triassic salt tectonics in the central NCA is, as discussed above and illustrated with Fig. 6, abundant. Salt most likely played a significant role in the configuration of the Middle to Upper Triassic carbonate platforms, controlling minibasin subsidence and tilting, thereby conditioning stratal geometries and thickness.

Inherited thickness changes in the platform carbonates and the presence of intra-platform salt structures most certainly played a significant role in the development of structures from Jurassic times onwards. The likely re-activation of salt structures during the Cretaceous emplacement of the Dachstein thrust sheet and the development of the Neogene KLT fault along pre-existing salt structures are two examples documented in this contribution. It is likely that further exploration can clarify the exact role played by inheritance and also bring to light new instances of structural re-activation of salt structures.

### Structure of the Sattelberg in the Lammertal zone

**Fig. 15:**
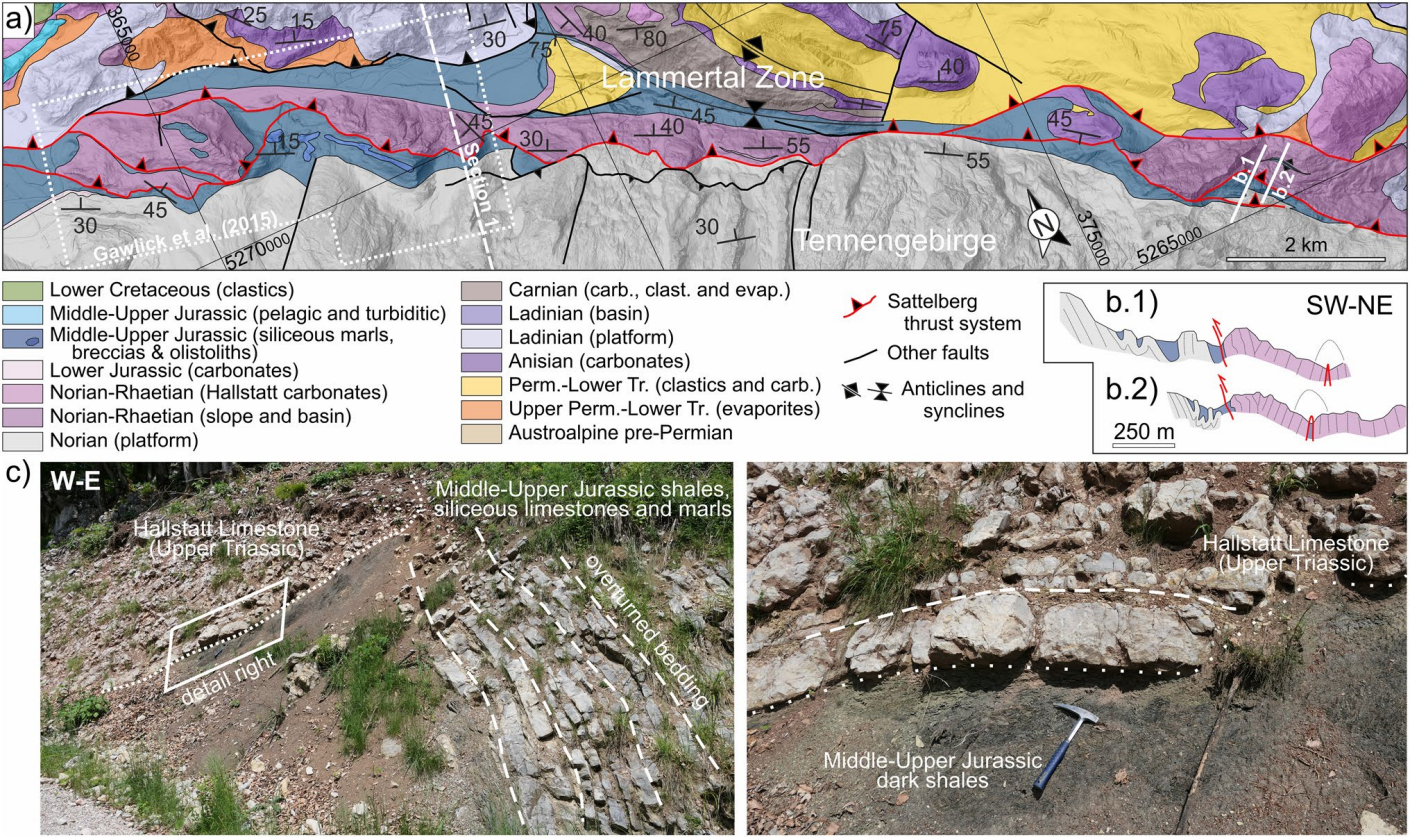
**a** Sub-quaternary geological map of the contact between the Lammertal Zone and the Tennengebirge interpreted in line with a scenario of relative autochthony. The contact between deep-water Triassic units of the Lammertal Zone and shallow-water carbonates of the Tennengebirge is interpreted to be a thrust system (Sattelberg thrust system). The map is adapted, with own observations, from Cornelius and Plöchinger (1952), Plöchinger (1982, 1987), and Gawlick and Missoni (2015), whose mapped area is shown. **b** Local cross-sections from Cornelius and Plöchinger (1952) across the eastern part of the Sattelberg thrust system showing south-vergent folding in the footwall of the Sattelberg thrust and a folded thrust surface (the thrust has been added to the original interpretation). **c** Example of Hallstatt Limestone (Upper Triassic) olistolith within Middle to Upper Jurassic shales and siliceous marls. It is noted that the contact between both has not recorded tectonic deformation. The detail photo on the right is roughly 2 m across. Location of outcrop: × 369,580, y 5,272,270 (WGS84, UTM33)

The Lammertal Zone is one of the key areas used to support the allochthonous origin of the Hallstatt units (e.g., Gawlick 1998; Gawlick and Missoni 2015, 2019). This area is characterized by the presence of both deep-water Hallstatt units and Middle to Upper Jurassic deep-water silica-rich shales and siliceous marls and limestones (Plöchinger 1987; Gawlick 1996, 1998; Gawlick and Missoni 2015) (Fig. 15a). Of particular relevance to this contribution is the nature of the contact between the deep-water Triassic units of the Lammertal Zone and their shallow-water equivalents of the Tennengebirge. Gawlick (1996, 1998) and Gawlick and Missoni (2015) document the presence of Jurassic breccias with an Upper Jurassic shale matrix in the northwestern end of the Lammertal (boxed area in Fig. 15a) and interpreted that blocks of Triassic deep-water rocks are olistoliths. The contacts between Upper Triassic deep-water carbonates and Upper Jurassic units, however, are often not observable due to outcrop conditions, but are locally well exposed. Some blocks of Triassic basinal carbonates, tens of meters in dimension, have exposed contacts with surrounding Jurassic shales that are sedimentary in nature, as expected for olistoliths (e.g., Fig. 15c). However, some contacts between deep-water Triassic and the Jurassic shales are locally observed to be tectonic. At the southeastern end of the Lammertal, Upper Triassic slope carbonates are found structurally above (to the NE in cross-section) the shallow-water Upper Triassic and Middle to Upper Jurassic shales of the Tennengebirge (Fig. 15b). There, the shallow-water carbonates and overlying Upper Jurassic shales are tightly folded into south-vergent anticlines and synclines (Cornelius and Plöchinger 1952; Fig. 15b). This is consistent with the south-directed thrusting reported by Schweigl and Neubauer (1997) for the deep-water Triassic of the Lammertal Zone.

The larger blocks of Triassic deep-water carbonates mapped by Plöchinger (1987) in the northwestern Lammertal and interpreted by Gawlick and Missoni (2015) to be kilometer-sized olistoliths (see the three fault-bounded blocks in the area mapped by those authors in Fig. 15a) are potentially part of a coherent system of backthrusts that runs along almost the entire length of the Lammertal (Fig. 15a).

Surprisingly, some of the Upper Triassic Lammertal Zone carbonates involved in the Sattelberg thrusts have been described as shallow-water carbonates (Cornelius and Plöchinger 1952; Plöchinger 1982) and could be part of a tectonically dismembered transition. A more detailed understanding of the Triassic facies distribution in the Lammertal Zone and, ideally, the identification of unambiguous contacts between units likely hold the key to determining the precise nature of the structure in this area.

### The Mandlingzug

As mentioned above, an aspect of post-Triassic deformation not specifically addressed in the restorations is the role of Neogene strike-slip tectonics. The most relevant effect of strike-slip observable on the cross-sections is the juxtaposition of the Mandlingzug against the southern end of the Dachstein unit (Fig. 8). The absence of a well-developed Upper Triassic succession on the Mandlingzug (Fig. 8) has led some authors to propose that this unit is highly allochthonous (e.g., Frisch and Gawlick 2003), while others interpreted it to be in continuation with the Dachstein unit (Neubauer 2016). A similar stratigraphic arrangement (a condensed to absent Upper Triassic) has been documented by Santolaria et al (2022), Strauss et al. (2023), and Oravecz et al. (2023) for some Triassic platform blocks in the eastern NCA and Western Carpathians. Santolaria et al. (2022) and Strauss et al. (2023) have explained the incomplete stratigraphy of these anomalous platform blocks as the result of drowning of the distal platform unit due to salt-detached downslope gliding. This mechanism has not been explored here, but could provide a reasonable explanation, implying a reduced allochthony of the Mandlingzug.

### Shortening estimates

Accurate estimates of shortening cannot be made on the cross-sections presented because of the likely prominence of out-of-plane motion. However, the relative autochthony scenario sections consistently estimate a shortening that is 20–50% less than in the allochthony scenario (Figs. 10, 11, 12). This order of magnitude is comparable to the estimates of Zankl (1967), who comparing his work with that of Spengler (1956) stated that shortening with a scenario of relative autochthony of the Hallstatt units would yield approximately half as much shortening as a scenario of Hallstatt unit allochthony.

### Metamorphism

A constraint on the tectonic evolution of the NCA that has not been fully explored are data on metamorphism. In the central NCA, Cretaceous widespread low grade metamorphism has been recorded in the Lammertal Zone and the southern end of the NCA (Kralik et al. 1987; Frank and Schlager 2006) (Fig. 13b). It can be inferred from this, that during the Cretaceous the trailing edge of the NCA was tectonically buried below units that have since been eroded (Fig. 13b). Some of these units might have comprised NCA-style stratigraphy, which would imply a wider southward extent of the Triassic NCA platform-and-basin domain. This would raise the question if the Upper Triassic platform-basin transition of the Gosaukamm (Kenter and Schlager 2009) was really the southern margin of the NCA platforms or a platform margin facing an intra-platform seaway.

Record of Cretaceous epimetamorphism (< 250 °C) also appears in locations farther north, specifically in association to Permo-Triassic evaporite diapirs (Kralik et al. 1987; Leitner et al. 2013). Kralik et al. (1987) argue in favor of a shallow origin for the epimetamorphic signal in the diapirs. This epimetamorphism contrasts with the limited amount of burial in parts of the Lammertal and in the footwall of the Dachstein thrust sheet indicated by the limited diagenesis experienced by Triassic strata, and Ar^40^/Ar^39^ and AFT data (Fig. 13b), consistent with documented temperatures below 150 °C since the Jurassic (e.g., Hejl and Grundmann 1989; Spötl and Hasenhüttl 1998; Mette et al. 2019). Explaining the origin of the complex post-Jurassic (and post-emplacement) metamorphic signals recorded in diapirs throughout the NCA is a problem that remains unsolved irrespective of the scenario interpretation.

### Implications for the structure of the central NCA

The structural analysis and cross-sections presented herein provide insights into the structure of the central NCA. For instance, the restoration of Section 2 (Fig. 11d, h) shows a contrast in stratigraphic thickness from the Langbathseen to the Höllengebirge unit. However, hardly any difference is observed between the Höllengebirge–Singereben and Dachstein units (Fig. 11d, h). It is therefore proposed here that the Dachstein thrust recorded a limited amount of displacement, likely in the order of 5–15 km. Furthermore, we propose its correlation eastward to the Warscheneck thrust sheet and westward to the Untersberg thrust sheet based on similar ages of emplacement, and potentially coherent NE-directed thrusting. In this scheme the Postalm and Salzsteig faults would have acted as lateral ramps to the system and the Schwarzer Berg and Tauplitzalm would have acted as transpressive oblique ramps during the Cretaceous (Fig. 5a).

In addition, we highlight the presence of two main belts of Jurassic shortening-related structures that cut across the entire area (Fig. 5a). A northern belt is formed by the east–west to northeast–southwest trending Totengebirge thrust and Trattberg Rise (a trend partly overthrust by the Dachstein thrust sheet) (Fig. 5a). The Totengebirge structure forms the southern limb of the Grünberg syncline (and northern margin of the Altaussee Zone; Tollmann 1976b) and extends beyond the Trattberg to the Eckersattel thrust (Fig. 5a, b.3). A southern belt of Jurassic structures corresponds to the contact of the Lammertal and Bad Mitterndorf Zones on the underlying Tennengebirge and Dachstein–Grimming shallow-water carbonates. These contacts are laterally continuous and named here the Sattelberg and Mitterndorf thrusts (Fig. 5a). The Sattelberg thrust extends westwards into the Bluntautal thrust (Fig. 5a). As discussed above, these are all potentially south-directed thrusts (backthrusts). This southern belt also includes shortening in the Hallstatt and Wurzeralm diapirs (Fig. 5a). In both cases, contacts of salt on Jurassic strata have been interpreted as the result of shortening-driven salt allochthony (Fernandez et al. 2021; Kurz et al. 2023).

### Open questions

Other than the aspects that need to be further explored to test the alternative scenarios for the stratigraphic and structural development of the NCA, there are a number of more general questions that arise from the work presented herein:

1. What was the direction of tectonic shortening within the central NCA during the Late Jurassic and Early Cretaceous? What was the role of the Austroalpine basement during these phases?
2. How does salt-related structural inheritance impact the interpretation of Cretaceous to Neogene structures?

## Conclusion: one geology, two interpretations

The cross-sections and restorations shown in this contribution aim to re-cast the debate on the origin of the Hallstatt units from a modern structural geological perspective. Two alternative and strongly contrasting scenarios are presented by means of cross-sections constrained by data from wellbores and outcrops. A scenario of allochthony of the Hallstatt units is based on the assumption that the Triassic facies belts along the NCA passive margin graded laterally from a single shallow-water platform in the North (Hauptdolomit–Dachsteinkalk megabank) into a single deep-water Hallstatt basin in the south. During the Middle to Late Jurassic, the basinal Hallstatt units were subject to northward long-distance transport, either by gravitational gliding or incorporated in an accretionary prism. A scenario of relative autochthony of the Hallstatt units is based on the assumption of Triassic salt tectonism in the central NCA, with the Hallstatt units deposited in subsiding, salt-floored seaways between partially isolated Triassic carbonate platforms.

Irrespective of the validity of each scenario, the sections and restorations presented here raise major questions about the traditional structural understanding of the central NCA and provide a modern structural framework within which to conduct future research.

## Data Availability

Geological maps used for cross-section construction are openly available from www.geologie.ac.at. Field dip data and revised mapping are available from the corresponding author upon request.

## References

[CR1] Aubert O, Droxler AW (1996). Seismic stratigraphy and depositional signatures of the Maldive carbonate system (Indian Ocean). Mar Petr Geol.

[CR2] Baumgartner P (2013). Mesozoic radiolarites—accumulation as a function of sea surface fertility on Tethyan margins and in ocean basins. Sedimentology.

[CR3] Bechstädt T, Schweizer T (1991). The carbonate-clastic cycles of the East-Alpine Raibl group: result of third-order sea-level fluctuations in the Carnian. Sed Geol.

[CR4] Bertotti G, Picotti V, Bernoulli D, Castellarin A (1993). From rifting to drifting: tectonic evolution of the South-Alpine upper crust from the Triassic to the Early Cretaceous. Sed Geol.

[CR5] Betzler C, Hübscher C, Lindhorst S, Reijmer JJG, Römer M, Droxler AW, Fürstenau J, Lüdmann T (2009). Monsoon-induced partial carbonate platform drowning (Maldives, Indian Ocean). Geology.

[CR6] Betzler C, Lindhorst S, Lüdmann T, Weiss B, Wunsch M, Braga JC (2015). The leaking bucket of a Maldives atoll: implications for the understanding of carbonate platform drowning. Mar Geol.

[CR7] Brandner R, Poleschinski W (1986). Stratigraphie und Tektonik am Kalkalpensüdrand zwischen Zirl und Seefeld in Tirol (Exkursion D am 3. April 1986). Jahresberichte Und Mitteilungen Des Oberrheinischen Geologischen Vereines Neue Folge.

[CR8] Braun R (1998) Die Geologie des Hohen Gölls. Nationalpark Berchtesgaden Forschungsbericht 40/1998, Berchtesgaden

[CR9] Brix F, Schultz O (1993). Erdöl und Erdgas in Österreich.

[CR10] Brückl E, Bleibinhaus F, Gosar A, Grad M, Guterch A, Hrubcová P, Keller GR, Majdański M, Šumanovac F, Tiira T, Yliniemi J, Hegedűs E, Thybo H (2007). Crustal structure due to collisional and escape tectonics in the Eastern Alps region based on profiles Alp01 and Alp02 from the ALP 2002 seismic experiment. J Geophys Res.

[CR11] Cornelius HP, Plöchinger B (1952). Der Tennengebirgs-N-Rand mit seinen Manganerzen und die Berge im Bereich des Lammertales. Jahrb Geol Bundesanst.

[CR12] Decker K, Peresson H, Faupl P (1994). Die miozäne Tektonik der östlichen Kalkalpen: Kinematic, Paläospannungen und Deformationsaufteilung während der “lateralen Extrusion” der Zentralalpen. Jahrb Geol Bundesanst.

[CR13] Diener C (1926). Die Fossillagerstätten in den Hallstätter Kalken des Salzkammergutes. Sitzungsberichte der Akademie der Wissenschaften in Wien, Mathematisch-naturwissenschaftliche Klasse. Abteilung I.

[CR14] Donofrio D, Brandner R, Poleschinski W (2003). Conodonten der Seefeld-Formation: Ein Beitrag zur Bio- und Lithostratigraphie der Hauptdolomit-Plattform (Obertrias, westliche Nördliche Kalkalpen, Tirol). Geologisch-Paläontologische Mitteilungen Innsbruck.

[CR15] Duffy OB, Dooley TP, Hudec MR, Jackson MPA, Fernandez N, Jackson CA-L, Soto JI (2018). Structural evolution of salt-influenced fold-and-thrust belts: a synthesis and new insights from basins containing isolated salt diapirs. J Struct Geol.

[CR16] Eberli GP, Ginsburg RN (1987). Segmentation and coalescence of Cenozoic carbonate platforms, northwestern Great Bahama Bank. Geology.

[CR17] Egger H (1996). Geologische Karte der Republik Österreich 1:50,000 Blatt 66 Gmunden.

[CR18] Egger H (2007). Erläuterungen zu Blatt 66 Gmunden.

[CR19] Egger H, van Husen D (2003). Geologische Karte der Republik Österreich 1:50,000 Blatt 64 Strasswalchen.

[CR20] Egger H, van Husen D (2007). Geologische Karte der Republik Österreich 1:50,000 Blatt 67 Grünau.

[CR21] Egger H, Heinrich M, van Husen D, Lobitzer H, Moshammer B, Pavuza R, Rupp C, Schedl A, Schubert G, Schuster R, Stummer G, Wagner L, Wessely G (2009). Erläuterungen zu Blatt 67 Grünau im Almtal.

[CR22] Egger H, Briguglio A, Rögl F (2017). Eocene straigraphy of the Reichenhall basin (Eastern Alps, Austria, Germany). Newsl Stratigr.

[CR23] Faupl P, Wagreich M (2000). Late Jurassic to Eocene paleogeography and geodynamic evolution of the Eastern Alps. Mitt Österr Geol Ges.

[CR24] Fernandez O, Habermüller M, Grasemann B (2021). Hooked on salt: Rethinking Alpine tectonics in Hallstatt (Eastern Alps, Austria). Geology.

[CR25] Fernandez O, Grasemann B, Sanders D (2022) Deformation of the Dachstein Limestone in the Dachstein thrust sheet (Eastern Alps, Austria). AJES 115:167–190. 10.17738/ajes.2022.0008

[CR26] Frank W, Schlager W (2006). Jurassic strike slip versus subduction in the Eastern Alps. Int J Earth Sci.

[CR27] Frisch W, Gawlick HJ (2003). The nappe structure of the central Northern Calcareous Alps and its disintegration during Miocene tectonic extrusion—a contribution to understanding the orogenic evolution of the Eastern Alps. Int J Earth Sci.

[CR28] Frisch W, Kuhlemann J, Dunkl I, Brügel A (1998). Palinspastic reconstruction and topographic evolution of the Eastern Alps during the late Tertiary tectonic extrusion. Tectonophysics.

[CR29] Froitzheim N, Plašienka D, Schuster R, McCann T (2008). Alpine tectonics of the Alps and Western Carpathians. The geology of central Europe.

[CR30] Froitzheim N, Weber S, Nagel TJ, Ibele T, Furrer H (2012). Late Cretaceous extension overprinting a steep belt in the Northern Calcareous Alps (Schesaplana, Rätikon, Switzerland and Austria). IJES.

[CR31] Gawlick H-J (1996). Die früh-oberjurassischen Brekzie der Strubbergschichten im Lammertal—Analyse und tektonische Bedeutung (Nördliche Kalkalpen, Österreich). Mitt Ges Geol Bergbaustud Österr.

[CR32] Gawlick H-J (1998). Obertriassische Brekzienbildung und Schollengleitung im Zlambachfaziesraum (Pötschenschichten)—Stratigraphie, Paläogeographie und diagenetische Überprägung des Lammeregg-Schollenkomplexes (Nördliche Kalkalpen, Salzburg). Jahrb Geol Bundesanst.

[CR33] Gawlick H-J (2000). Sedimentologie, Fazies und Stratigraphie der obertriassischen Hallstätter Kalke des Holzwehralm-Schollenkomplexes (Nördliche Kalkalpen, Salzburger Land). Jahrb Geol Bundesanst.

[CR34] Gawlick H-J, Missoni S (2015). Middle Triassic radiolarite pebbles in the Middle Jurassic Hallstatt Mélange of the Eastern Alps: implications for Triassic-Jurassic geodynamic and paleogeographic reconstructions of the western Tethyan realm. Facies.

[CR35] Gawlick H-J, Missoni S (2019). Middle-Late Jurassic sedimentary mélange formation related to ophiolite obduction in the Alpine-Carpathian-Dinaridic Mountain Range. Gondwana Res.

[CR36] Gawlick H-J, Frisch W, Vecsei A, Steiger T, Böhm F (1999). The change from rifting to thrusting in the Northern Calcareous Alps as recorded in Jurassic sediments. Geol Rundsch.

[CR37] Gawlick H-J, Schlagintweit F, Suzuki H (2007). Die Ober-Jura bis Unter-Kreide Schichtfolge des Gebiets Höherstein-Sandling (Salzkammergut, Österreich)—Implikationen zur Rekonstruktion des Block-Puzzles der zentralen Nördlichen Kalkalpen, der Gliederung der Radiolaritflyschbecken und der Plassen-Karbonatplattform. N Jb Gelo Paläont Abh.

[CR38] Gawlick H-J, Missoni S, Schlagintweit F, Suzuki H, Frisch W, Krystyn L, Blau J, Lein R (2009). Jurassic tectonostratigraphy of the Austroalpine Domain. Jour Alpine Geol.

[CR39] Geutebrück E, Klammer W, Schimunek K, Steiger E, Ströbl E, Winkler G, Zych D (1984). Oberflächengeophysikalische Verfahren im Rahmen der KW-exploration der ÖMV. Erdöl Und Erdgas.

[CR40] Granado P, Roca E, Strauss P, Pelz K, Muñoz JA (2019). Structural styles in fold-and-thrust belts involving early salt structures: the Northern Calcareous Alps (Austria). Geology.

[CR41] Griffiths P, Jones S, Salter N, Schaefer F, Osfield R, Reiser H (2002). A new technique for 3-D flexural-slip restoration. J Struct Geol.

[CR42] Haas J, Kovács S, Krystyn L, Lein R (1995). Significance of Late Permian-Triassic facies zones in terrane reconstructions in the Alpine-North Pannonian domain. Tectonophysics.

[CR43] Hahn FF (1913). Grundzüge des Baues der nördlichen Kalkalpen zwischen Inn und Enns. I Teil Mitt Österr Geol Ges.

[CR44] Hahn FF (1913). Grundzüge des Baues der nördlichen Kalkalpen zwischen Inn und Enns. II Teil Mitt Österr Geol Ges.

[CR45] Hamilton W (1989). Geologische Ergebnisse von Tiefbohrungen im Flysch und Kalkalpin zwischen Wien und Salzburg: Exkursionsführer der Österreichischen Geologischen Gesellschaft Nr 12.

[CR46] Häusler H (1980). Zur tektonischen Gliederung der Lammer-Hallstätter Zone zwischen Golling und Abtenau (Salzburg). Mitt Österr Geol Ges.

[CR47] Häusler H, Berg D (1980). Neues zur Stratigraphie und Tektonik der Hallstätter Zone am Westrand der Berchtesgadener Masse. Verh Geol B-A.

[CR48] Héja G, Kövér S, Csillag G, Németh A, Fodor L (2018). Evidences for pre-orogenic passive-margin extension in a Cretaceous fold-and-thrust belt on the basis of combined seismic and field data (western Transdanubian Range, Hungary). IJES.

[CR49] Hejl E, Grundmann G (1989). Apatit-Spaltspurdaten zur thermischen Geschichte der Nördlichen Kalkalpen, der Flysch- und Molassezone. Jahresb Geol Bundesanst.

[CR50] Hetényi G, Polmerová J, Bianchi I, Kampfová Exnerová H, Bokelmann G, Handy MR, Babuška V, AlpArray-EASI Working Group (2018). From mountain summits to roots: Crustal structure of the Eastern Alps and Bohemian Massif along longitude 13.3°E. Tectonophysics.

[CR51] Hine A, Wilber R, Neumann A (1981). Carbonate sand bodies along contrasting shallow bank margins facing open seaways in northern Bahamas. AAPG Bull.

[CR52] Hornung T (2007). Multistratigraphy of the Draxllehen quarry near Berchtesgaden (Tuvalian-Lacian 2): Implications for Hallstatt limestone sedimentation and palaeoclimate in the aftermath of the 'Carnian Crisis'. AJES.

[CR53] Hornung T, Spatzenegger A, Joachimski MM (2007). Multistratigraphy of condensed ammonoid beds of the Rappoltstein (Berchtesgaden, southern Germany): unravelling palaeoenvironmental conditions on ‘Hallstatt deep swells’ during the Reingraben Event (Late Lower Carnian). Facies.

[CR54] Jackson MPA, Hudec MR (2017). Salt tectonics: principles and practice.

[CR55] Kenter JAM, Schalger W (2009). Slope angle and basin depth of the Triassic platform-basin transition at the Gosaukamm, Austria. AJES.

[CR56] Kralik M, Krumm H, Schramm JM, Flügel HW, Faupl P (1987). Low grade and very low grade metamorphism in the Northern Calcareous Alps and in the Greywacke Zone: illite-crystallinity data and isotopic ages. Geodynamics of the Eastern Alps.

[CR141] Kilian S, Ortner H (2019). Structural evidence of in-sequence and out-of-sequence thrusting in the Karwendel mountains and the tectonic subdivision of the western Northern Calcareous Alps. AJES.

[CR57] Krenmayr HG (2005). Geologische Karte von Salzburg 1:200,000.

[CR58] Krenmayr HG (2009). Zusammenstellung ausgewählter Archivunterlagen der Geologischen Bundesanstalt GEOFAST 1:50,000 Blatt 93 Bad Reichenhall.

[CR59] Krenmayr HG (2013). Geologische Karte der Republik Österreich 1:50,000 Blatt 125 Bischofshofen.

[CR60] Krenmayr HG, Schnabel H-J (2006). Geologische Karte von Oberösterreich 1:200,000.

[CR61] Kreuss O (2020). Zusammenstellung ausgewählter Archivunterlagen der Geologischen Bundesanstalt GEOFAST 1:50,000 Blatt 128 Gröbming.

[CR62] Krische O, Gawlick H-J (2015) Age and significance of Lower Cretaceous mass flows: Ischl Breccia revisited (Rossfeld Formation, Northern Calcareous Alps, Austria). AJES 108:128–150. 10.17738/ajes.2015.0017

[CR63] Kurz M, Fernandez O, Eggerth L, Grasemann B, Strauss P (2023) Emplacement and associated sedimentary record of the Jurassic submarine salt allochthon of the Wurzeralm (Eastern Alps, Austria). Terra Nova. 10.1111/ter.12675 10.1111/ter.12675 PMC10958983 38524903

[CR64] Leitner C, Spötl C (2017) The eastern Alps: Multistage development of extremely deformed evaporites. In: Soto JI, Flinch JF, Tari G (eds) Permo-Triassic salt provinces of Europe, North Africa and the Atlantic margins. Tectonics and hydrocarbon potential. Elsevier, Amsterdam, pp 467–482. 10.1016/B978-0-12-809417-4.00022-7

[CR65] Leitner C, Neubauer F, Genser J, Borojević-Šoštarić S, Rantitsch G (2013) ^40^Ar/^39^Ar ages of crystallization and recrystallization of rock-forming polyhalite in Alpine rocksalt deposits. In: Jourdan F, Mark DF, Verati C (eds) Advances in 40Ar/39Ar Dating: from Archaeology to Planetary Sciences. Geological Society, London, Spec Pub, vol 378, pp 207–224. 10.1144/SP378.5

[CR66] Levi N (2023). Polyphase tectonics in the central Salzkammergut (Northern Calcareous Alps, Austria): An updated interpretation. Jour Geodyn.

[CR67] Levi N, Pittarello L, Habermüller M (2022). Structural characteristics of the curved Königsee-Lammertal-Traunsee fault system in Salzkammergut (Northern Calcareous Alps, Austria). J Struct Geol.

[CR68] Lingrey S, Vidal-Royo O (2015) Evaluating the quality of bed length and area balance in 2D structural restorations. Interpretation 3:SAA133–SAA160. 10.1190/INT-2015-0126.1

[CR69] Linzer H-G, Ratschbacher L, Frisch W (1995). Transpressional collision structures in the upper crust: the fold-thrust belt of the Northern Calcareous Alps. Tectonophysics.

[CR70] Linzer H-G, Decker K, Peresson H, Dell’Mour R, Frisch W (2002). Balancing lateral orogenic float of the Eastern Alps. Tectonophysics.

[CR71] Lüdmann T, Kalvelage C, Betzler C, Fürstenau J, Hübscher C (2013). The Maldives, a giant isolated carbonate platform dominated by bottom currents. Mar Petr Geol.

[CR72] Lüdmann T, Paulat M, Möbius J, Lindhorst S, Wunsch M, Eberli GP (2016). Carbonate mounds in the Santaren Channel, Bahamas: a current-dominated periplatform depositional regime. Mar Geol.

[CR73] Mandl GW (1984). Zur Trias des Hallstätter Faziesraumes—ein Modell am Beispiel Salzkammergut (Nördliche Kalkalpen, Österreich). Mitt Ges Geol Bergbaustud Öst.

[CR74] Mandl GW (1998). Geologische Karte der Dachsteinregion 1:50,000.

[CR75] Mandl GW (2000). The Alpine sector of the Tethyan shelf—Examples of Triassic to Jurassic sedimentation and deformation from the North Calcareous Alps. Mitt Österr Geol Ges.

[CR76] Mandl GW (2003). Hallstätter Kalke auf dem Sarstein? (Salzkammergut, Oberösterreich). Jahrb Geol Bundesanst.

[CR77] Mandl GW (2013). Zur Geologie des Raumes Hütteneckalm–Sandlingalm–Blaa-Alm (Salzkammergut, Österreich) mit kritischen Anmerkungen zur Sandlingalm-Formation. Jahrb Geol Bundesanst.

[CR78] Mandl GW, Matura A (1995). Geologische Karte der Republik Österreich 1:50,000 Blatt 127 Schladming.

[CR79] Mandl GW, van Husen D, Lobitzer H (2012). Erläuterung zu Blatt 96 Bad Ischl.

[CR80] Mandl GW, Hejl E, van Husen D (2014). Erläuterung zu Blatt 127 Schladming.

[CR81] McRoberts CA, Krystyn L, Shea A (2008). Rhaetian (Late Triassic) Monotis (Bivalvia: Petinoida) from the eastern Northern Calcareous Alps (Austria) and the end-Norian crisis in pelagic faunas. Palaeontology.

[CR82] Medwenitsch W, Schauberger O (1951) Hallstätter Salzberg. Verh Geol BA Sonderheft A:Tafel X

[CR83] Mette W, Clemence M-E, Thibault N, Korte C, Konrad B, Ullman CV (2019). Sedimentology, carbon isotope stratigraphy and micropalaeontology of the Rhaetian Zlambach Formation—implications for the Dachstein carbonate platform development (Northern Calcareous Alps, Austria). Sed Geol.

[CR84] Miladinova I, Froitzheim N, Nagel TJ, Janák M, Fonseca ROC, Sprung P, Münker C (2022). Constraining the process of intracontinental subduction in the Austroalpine Nappes: implications from petrology and Lu-Hf geochronology of eclogites. Jour Metam Geol.

[CR85] Missoni S, Gawlick H-J (2011). Evidence for Jurassic subduction from the Northern Calcareous Alps (Berchtesgaden; Austroalpine, Germany). Int J Earth Sci.

[CR86] Moser M (2014). Zusammenstellung ausgewählter Archivunterlagen der Geologischen Bundesanstalt GEOFAST 1:50,000 Blatt 97 Bad Mitterndorf.

[CR87] Neubauer F (2016). Formation of an intra-orogenic transtensional basin: the Neogene Wagrain basin in the Eastern Alps. Swiss Jour Geosci.

[CR88] Neubauer F, Genser J (2018). Field Trip Post-EX-1—transect across the Eastern Alps. Berichte Der Geologischen Bundesanstalt (österreich).

[CR89] Ogg J (2015). The mysterious mid-Carnian “Wet Intermezzo” global event. Jour Earth Sci.

[CR90] Oravecz É, Héja G, Fodor L (2023). Salt tectonics versus shortening: Recognizing pre-orogenic evaporite deformation in salt-bearing fold-and-thrust belts on the example of the Silica Nappe (Inner Western Carpathians). Tectonics.

[CR91] Ortner H (2017) Geometry of growth strata in wrench-dominated transpression: 3D-model of the Upper Jurassic Trattberg rise, Northern Calcareous Alps, Austria. Geophys Res Abstr 19:EGU2017–9222

[CR92] Ortner H, Kilian S (2022). Thrust tectonics in the Wetterstein and Mieming mountains, and a new tectonic subdivision of the Northern Calcareous Alps of Western Austria and Southern Germany. Int J Earth Sci.

[CR93] Ortner H, Stingl V (2001) Facies and basin development of the Oligocene of the Lower Inn Valley, Tyrol/Bavaria. In: Piller W, Raser M (eds) Paleogene in Austria. Schriftenreihe der Erdwissenschaftlichen Komissionen 14, ÖAW, Vienna, pp 153–196

[CR94] Ortner H, Ustaszewski M, Rittner M (2008). Late Jurassic tectonics and sedimentation: breccias in the Unken syncline, central Northern Calcareous Alps. Swiss J Geosci.

[CR95] Ortner H, Ganser C, Stipp M, Fernandez O (2022). Deformation of a mountain-sized olistolith: Schwarzer Berg, Northern Calcareous Alps of Salzburg. Berichte Der Geologischen Bundesanstalt.

[CR96] Pavlik W (2007). Provisorische Geologische Karte der Republik Österreich GEOFAST 1:50,000 Blatt 92 Lofer.

[CR97] Pavlik W (2009). Zusammenstellung ausgewählter Archivunterlagen der Geologischen Bundesanstalt GEOFAST 1:50,000 Blatt 93 Bad Reichenhall.

[CR98] Pavlik W (2013a) Zusammenstellung ausgewählter Archivunterlagen der Geologischen Bundesanstalt GEOFAST 1:50,000 Blatt 124 Saalfelden a. Stein. Meer. Geologische Bundesanstalt, Vienna

[CR99] Pavlik W (2013). Zusammenstellung ausgewählter Archivunterlagen der Geologischen Bundesanstalt GEOFAST 1:50,000 Blatt 125 Bischofshofen.

[CR100] Pavlik W (2014). Zusammenstellung ausgewählter Archivunterlagen der Geologischen Bundesanstalt GEOFAST 1:50,000 Blatt 98 Liezen.

[CR101] Peresson H, Decker K (1997). The Tertiary dynamics of the northeastern Eastern Alps (Austria): changing paleostresses in a collisional plate boundary. Tectonophysics.

[CR102] Pistotnik U (1972). Zur Mikrofazies und Paläogeographie der Zlambachschichten (O. Nor—? U. Lias) im Raume Bad Goisern—Bad Aussee (Nördliche Kalkalpen). Mitt Ges Geol Bergbaustud.

[CR103] Pistotnik U (1974). Fazies und Tektonik der Hallstätter Zone von Bad Ischl—Bad Aussee (Salzkammergut, Österreich). Mitt Geol Ges Wien.

[CR104] Plašienka D (2018). Continuity and episodicity in the early Alpine tectonic evolution of the Western Carpathians: How large-scale processes are expressed by the orogenic architecture and rock record data. Tectonics.

[CR105] Plöchinger B (1964). Die tektonischen Fenster von St. Gilgen und Strobl am Wolfgangsee (Salzburg, Oberösterreich). Jahrb Geol Bundesanst.

[CR106] Plöchinger B (1982). Geologische Karte der Republik Österreich 1:50,000 Blatt 95 Sankt Wolfgang.

[CR107] Plöchinger B (1987). Geologische Karte der Republik Österreich 1:50,000 Blatt 94 Hallein.

[CR108] Plöchinger B, Draxler I (1974). Gravitativ transportiertes permishes Haselgebirge in den Oberalmer Schichten (Tithonium, Salzburg). Verh Geol B-A.

[CR109] Ratschbacher L, Neubauer F (1989) West-directed decollement of Austro-Alpine cover nappes in the eastern Alps: geometrical and rheological considerations. In: Coward MP, Dietrich D, Parker RG (eds) Alpine tectonics, vol 45. Geological Society, London, Special Publication, pp 243–262

[CR110] Ratschbacher L, Frisch L, Linzer H-G (1991). Lateral extrusion in the Eastern Alps, Part 2: structural analysis. Tectonics.

[CR111] Rittner KM (2006) Geologie der östlichen Unkener Mulde am Kontakt zur Berchtesgadener Masse: Strukturgeologie und elektronische Verarbeitung geologischer Daten. Unpublished MSc thesis, University of Innsbruck

[CR112] Rowan MG (2017) An overview of allochthonous salt tectonics. In: Soto JI, Flinch JF, Tari G (2017) Permo-Triassic Salt Provinces of Europe, North Africa and the Atlantic Margins, pp 97–114. 10.1016/B978-0-12-809417-4.00005-7

[CR113] Rowan MG, Giles KA (2021). Passive versus active salt diapirism. AAPG Bull.

[CR114] Santolaria P, Granado P, Wilson E, de Matteis M, Ferrer O, Strauss P, Pelz K, König M, Oteleanu AE, Roca E, Muñoz JA (2022) From salt-bearing rifted margins to fold-and-thrust belts. Insights from analog modeling and Northern Calcareous Alps case study. Tectonics 41:e2022TC007503. 10.1029/2022TC007503

[CR115] Schäffer G (1982). Geologische Karte der Republik Österreich 1:50,000 Blatt 96 Bad Ischl.

[CR116] Schauberger O (1955). Zur Genese des alpinen Haselgebirges. Zeitschrift Der Deutschen Geologischen Gesellschaft.

[CR117] Schlager W (1967). Hallstätter und Dachsteinkalk-Fazies am Gosaukamm und die Vorstellung ortsgebundener Hallstätter Zonen in den Ostalpen. Verh Geol B-A.

[CR118] Schlager W (1969). Das Zusammenwirken von Sedimentation und Bruchtektonik in den triadischen Hallstätterkalken der Ostalpen. Geol Rund.

[CR119] Schlager W, Schöllnberger W (1974). Das Prinzip stratigraphischer Wende in der Schichtfolge der Nördlichen Kalkalpen. Mitt Geol Ges Wien.

[CR120] Schlagintweit F, Gawlick H-J (2006). Sarstenia babai n.gen., n. sp., a new problematic sponge (inozoa?) from the Late Jurassic of the Northern Calcareous Alps, Austria. Riv Ital Paleontol Stratigr.

[CR121] Schmid C, Mandl GW, Wessely G (2003). Thermalwasserbohrung Bad Mitterndorf TH 1: Ein kalkalpiner tiefenaufschluss im steirischen salzkammergut. Gmunder Geo-Studien.

[CR122] Schmid SM, Fügenschuh B, Kissling E, Schuster R (2004). Tectonic map and overall architecture of the Alpine orogen. Eclogae Geol Helv.

[CR123] Schmid SM, Bernoulli D, Fügenschuh B, Matenco L, Schefer S, Schuster R, Tischler M, Ustaszewski K (2008). The Alpine-Carpathian-Dinaridic orogenic system: correlation and evolution of tectonic units. Swiss J Geosci.

[CR124] Schöllnberger W (1973). Zur Verzahnung von Dachsteinkalk-Fazies und Hallstätter Fazies am Südrand des Toten Gebirges (Nördliche Kalkalpen, Österreich). Mitt Ges Geol Bergbaustud.

[CR125] Schöllnberger WE (2021). Comment on Strauss et al., 2021: Subsidence analysis of salt tectonics-driven minibasins (Northern Calcareous Alps, Austria). Basin Res.

[CR126] Schorn A, Neubauer F (2011). Emplacement of an evaporitic mélange nappe in central Northern Calcareous Alps: evidence from the Moosegg Klippe (Austria). AJES.

[CR127] Schorn A, Neubauer F (2014). The structure of the Hallstatt evaporite body (Northern Calcareous Alps, Austria): a compressive diapir superposed by strike-slip shear?. J Struct Geol.

[CR128] Schuster R, Egger H, Krenmayr HG, Linner M, Mandl GW, Matura A, Nowotny A, Pascher G, Pestal G, Pistotnik J, Rockenschaub M, Schnabel W (2015). Geologische Übersichtskarte der Republik Österreich 1:1,500,000 (Ohne Quartär).

[CR129] Schuster R, Daurer A, Krenmayr H-G, Linner M, Mandl GW, Pestal G, Reitner JM (2019). Rocky Austria: Geologie von Österreich—kurz und bunt.

[CR130] Schweigl J, Neubauer F (1997). Structural evolution of the central Northern Calcareous Alps: Significance of the Jurassic to Tertiary geodynamics of the Alps. Eclogae Geol Helv.

[CR131] Spengler E (1956). Versuch einer Reokonstruktion des Ablagerungsraumes der Decken der Nördlichen Kalkalpen, II. Teil: Der Mittelabschnitt der Kalkalpen. Jarhb Geol Bundesanst.

[CR142] Spötl C, Hasenhüttl C (1998). Thermal history of the evaporitic Haselgebirge mélange in the Northern Calcareous Alps (Austria). Geol Rundsch.

[CR132] Strauss P, Granado P, Muñoz JA (2021). Subsidence analysis of salt tectonics-driven carbonate minibasins (Northern Calcareous Alps, Austria). Bas Res.

[CR133] Strauss P, Granado P, Muñoz JA, Böhm K, Schuster R (2023). The Northern Calcareous Alps revisited: Formation of a hyperextended margin and mantle exhumation in the Northern Calcareous Alps sector of the Neo-Tethys (Eastern Alps, Austria). Earth-Sci Rev.

[CR134] Tollmann A (1960). Die Hallstätterzone des östlichen Salzkammergutes und ihr Rahmen. Jahrb Geol Budesanst.

[CR135] Tollmann A (1976a) Analyse des clasischen nordalpinen Mesozoikums: Stratigraphie, Fauna und Fazies der Nördlichen Kalkalpen. Franz Deuticke, Vienna

[CR136] Tollmann A (1976). Der Bau der Nördlichen Kalkalpen: Orogene Stellung und regionale Tektonik.

[CR137] Tollmann A (1981). Oberjurassische Gleittektonik als Hauptformungsprozeß der Hallstätter Region und neue Daten zur Gesamttektonik der Nördlichen Kalkalpen in den Ostalpen. Mitt Österr Geol Ges.

[CR138] Vendeville BC, Jackson MPA (1992). The fall of diapirs during thin-skinned extension. Mar Petr Geol.

[CR139] Wagreich M, Decker K (2001). Sedimentary tectonics and subsidence modelling of the type Upper Cretaceous Gosau basin (Northern Calcareous Alps, Austria). Int Jour Earth Sci.

[CR140] Zankl H (1967). Die Karbonatsedimente der Obertrias in den nördlichen Kalkalpen. Geol Rund.

